# Multimodal deep feature fusion with transformer for brain tumor classification from magnetic resonance imaging

**DOI:** 10.1038/s41598-026-44957-9

**Published:** 2026-04-11

**Authors:** M. Pajany, K. Boopalan, R. Rajesh, W. JaiSingh, Bibhuti Bhusan Dash, Saroja Kumar Rout, P. Pavan Kumar

**Affiliations:** 1 Department of Computer Science and Engineering, Jain Deemed To Be University, Bangalore, Karnataka 562112 India; 2 School of Computing, Vel Tech Rangarajan Dr. Sagunthala R&D Institute of Science and Technology, Avadi, Chennai, India; 3 Department of Computer Science and Engineering, Vel Tech Rangarajan Dr. Sagunthala R&D Institute of Science and Technology, Avadi, Chennai, 600062 India; 4 School of Computer Science and Applications, S-VYASA (Deemed to be University), Bengaluru, Karnataka 560059 India; 5 School of Computer Applications, Kalinga Institute of Industrial Technology (KIIT) Deemed to be University, Bhubaneswar, India; 6 School of Computer Science & Engineering, VIT-AP University, Amaravati, Andhra Pradesh India; 7 Department of AI&DS, ICFAITECH, Faculty of Science and Technology), IFHE, Hyderabad, India

**Keywords:** Brain Tumor, Segmentation, CapsNet, Transformer, ResNet-50, Deep Learning, Magnetic Resonance Imaging, Cancer, Computational biology and bioinformatics, Engineering, Mathematics and computing, Medical research

## Abstract

Brain tumors (BTs) arise due to abnormal cell growth, which has a high mortality rate globally. Millions of lives can be saved through the timely identification of BT. Precise identification and segmentation of BTs are essential to enhance the precision of analysis and the efficiency of therapeutic strategies. Magnetic resonance imaging (MRI) is a broadly utilized analytical tool. Furthermore, deep learning (DL) has recently shown efficiency in addressing several computer vision tasks. Several DL-driven methods are implemented for BT segmentation and attained impressive outcomes. This study presents a Multimodal Deep Feature Fusion Framework for Automated Brain Tumor Detection and Segmentation (MDFF-ABTDS) model. This objective is to develop a multimodal DL that integrates feature fusion and transformer networks for the precise detection and segmentation of BTs from medical images. Initially, image pre-processing is performed using Contrast Limited Adaptive Histogram Equalization (CLAHE) and image normalization. Feature extraction is carried out through fusion models such as CapsNet, ResNet-50, and AlexNet. These extracted features are then passed to a bi-directional convolutional long short-term memory combined with transformer (TBConvL-Net) models to classify tumors and non-tumors effectively. Finally, the tumor is classified to identify its location using the nnUNet model for a precise segmentation process. A series of experimental analyses of the MDFF-ABTDS method portrayed a superior accuracy value of 98.91% over existing models under the BT MRI dataset.

## Introduction

The brain acts as the governing point that controls nearly every vital activity through the processing of information and the sending of signals to the rest of the body^1^. It is considered responsible for movement, creativity, speech, thought, governing emotions, memory, intelligence, physical activity, senses, taste, etc. Therefore, some mishap or harm to the vital organ can disrupt the body’s proper functioning and overall routine^2^. It is critical for capturing the extreme care of these valuable organs. Among the diverse issues affecting the brain, the most frequent and life-threatening issues nowadays are those of BTs^3^. Each year, approximately 11,000 individuals are diagnosed with a BT. A BT is an anomalous lump of flesh caused by uncontrolled development and cell multiplication^4^. There are around 130 various kinds of BTs, and they are identified based on the cell type from which they originate, their growth rate, and their location. But the BTs are classified into two main classes: secondary BTs and primary BTs. The Primary BT originates from brain tissue or the tissue surrounding it^5^. A secondary BT forms when cancer cells migrate through the bloodstream. Therefore, timely diagnosis and accurate pinpointing of BTs are critically significant^6^. Diagnose image is the most helpful tool in medicine these days. MRI and computed tomography (CT) scans are the most often deployed diagnostic tools^2^. For BT recognition, precise segmentation is a critical procedure. BT segmentation divides a portion into mutually exclusive and populated areas^7^.

Therefore, the physical segmentation is time-consuming for the radiologist, and thus, semi-automated/automated models are needed for accurate tumor recognition. These days, a fully automated approach for identification among the non-tumor and tumor MRI is usual for research and medical examinations^8^. These approaches may offer more aid for analyzing the tumor area and have evolved quickly in the past ten years. However, radiologists assume that computerized approaches may enhance their diagnostic capabilities based on automating machine learning (ML) models^9^. Furthermore, with the assured performance generated by an effective DL approach, several DL-driven models have been used for BT segmentation to automatically feature extraction and obtain precise and steady performance. Numerous DL as well as ML techniques have been improved for several challenges in BT MRI imaging segmentation. Deep neural networks (DNNs), like U-Net, and convolutional neural networks (CNNs), have displayed huge success in BT MRI imaging segmentation by learning hierarchical characteristics and taking difficult patterns^10^. Several authors have obtained additional studies on DL models that depend on medical, i.e., employing CNNs for prediction, treatment planning, and disease diagnosis, etc. In addition, CNN has been employed for segmenting tumors in multimodal images^6^.

This study presents a Multimodal Deep Feature Fusion Framework for Automated Brain Tumor Detection and Segmentation (MDFF-ABTDS) approach. The contributions are summarized as follows:

- Development of a new MDFF-ABTDS approach for effective BT detection and segmentation using biomedical imaging.
- Implementation of CLAHE and image normalization for improved contrast and standardized image intensity.
- Multi-model CNN architectures of CapsNet, ResNet-50, and AlexNet for comprehensive spatial and contextual feature extraction.
- The TBConvL-Net model is designed to capture both spatial hierarchies and temporal dependencies for accurate classification.
- Use of nnUNet to achieve high-precision tumor localization and segmentation performance.
- Extensive comparative experiments prove that the MDFF-ABTDS outperforms existing benchmark models across multiple evaluation measures.

The paper is organized as follows: Sect.  2 reviews existing studies on BT segmentation. Section  3 explains the proposed methodology, including pre-processing, feature extraction, classification, and segmentation. Section  4 presents the experimental results. Finally, Sect.  5 concludes with key findings and contributions.

## Related works on BT detection and segmentation

This section presents a comprehensive review related to BT detection and segmentation techniques. Tanone et al.^11^ utilized a new structure termed ViT-CB that uses the ViT DL technique for feature extractors. Then, PCA is employed to improve the technique’s efficiency. The CatBoost model is additionally used for enhancing the technique’s last BT classifies performance. Moreover, uses SHAP-driven XAI for interpreting the techniques’ outcomes, classifying the characteristics that most influence the last identification. Rajendirane et al.^12^ proposed a complete method to improve BT detection in MRI imaging employing an integration of sophisticated models. Initially, an improved K-means clustering technique was used to segment tumor areas. Therefore, Median Filtering (MF) is used to refine imaging quality. The presented work is intended for improving the precision and effectiveness of BT recognition, providing potential developments in healthcare imaging diagnosis and analysis. Arshad Choudhry et al.^13^ explored the application of explainability models in BT segmentation employing MRI information. The adaptive learning class activation map (AL-CAM) uses a unique many-pop-out training approach and contrastive learning for improving internal outputs, enhancing interpretability. Moreover, a new method was proposed to explainability in graph CNNs (GCNNs). The utilization of conventional CNN interpretability tools is frequently unable to manage the difficulties of graph-structured information. Benedict et al.^14^ intended to identify and localize tumor regions in the brain using an image processing model and a deep wavelet autoencoder (DWAE) technique. Originally, a specialized clustering method was used for MRI imaging to segment the tumor regions. Then, the detected tumor areas are refined employing thresholding as well as level-set segmentation approaches for achieving precise delineation. Lastly, a DWAE is used for accurate identification of BT driven by the feature extraction. Khan et al.^15^ solved this problem by using data augmentation as well as denoising systems on health care images from 3 distinct sources of information, intending to improve detection effectiveness. For evaluating the efficiency of the approaches, they applied dual DL techniques employing CNNs that proved higher precision. These outcomes imply that integrating data augmentation and denoising models enhances the precision of BT diagnosis. Ali et al.^16^ presented a DL method integrating a Generative Adversarial Network (GAN) with transfer learning (TL) and autoencoder (AE) models for enhancing BT segmentation. The GAN integrates a discriminator and a generator to make a higher segmentation result. The generator used upsampling and downsampling for tumor segmentation. Moreover, an AE is used in which the encoder holds as much data as possible, and then the decoder, with those encodings, rebuilds the image. The TL method is used on the bottleneck employing the DenseNet technique. Allah et al.^17^ proposed a deep CNN (DCNN), termed the Edge U-Net technique. The Edge U-Net technique may localise tumors by integrating boundary-associated MRI with the major information from MRIs.

Rahman and Islam^18^ presented a new parallel deep CNN (PDCNN) topology for extracting either global or local characteristics from the dual parallel phases and dealing with the over-fitting issues by employing dropout regularization along with batch normalization. The advantage of parallel pathways is offered by integrating dual concurrent DCNNs with various window sizes, enabling this technique for learning global as well as local data. Yadav et al.^19^ segmented BT from MRI scans by employing DL methods, namely an EfficientNetB7-based UNet + + architecture with TL. Jenifer and Rajakumar^20^ utilized the HyperLink Bi-Attention U-Net with Transformer and Masked Autoencoder (HyBiUnet-TransMask) model. The Swin Transformer and Convolutional Neural Network (CNN) encoders, dual Bi-Attention mechanisms comprising Tumor-Region Context Attention (TRCA) and Channel Modality Relevance Attention (CMRA), and a Retrieval-Based Masked Autoencoder (RP-MaskAE) are also used for robust feature integration, cross-scanner generalization, and interpretable clinical decision support. Yadav, Kolekar, and Zope^21^ proposed the Modified Recurrent Residual Attention U-Net (Mod-R2AU-Net) model by employing Recurrent Residual Convolutional Layers and Attention Gates (AGs) to capture subtle features, refine important regions, and enhance segmentation accuracy. Rastogi et al.^22^ proposed a dual-stage DL methodology by integrating CNN models, namely MobileNet, NASNetMobile, and ResNet101, with a RESidual U-Net (RESUNET) segmentation network. The model also used TL to enhance feature extraction and generalization. Yadav et al.^23^ utilized advanced pre-processing techniques, multi-view image analysis, and a modified Visual Geometry Group-16 (VGG-16) model with TL. Yadav and Kolekar^24^ utilized DL methods comprising CNN, U-Net, and its extensions, attention mechanisms (AMs), and transformer-based architectures. Zhou et al.^25^ developed and validated Transformer-based DL models utilizing Transformer architectures. Maheshwari, Kelkar, and Francis^26^ utilized a hybrid DL method by incorporating CNN, ResNet18, and EfficientNetB0, with Vision Transformers (ViT) for enhanced feature extraction. Feature selection is further optimized by utilizing Particle Swarm Optimization (PSO) and Ant Colony Optimization (ACO). Furthermore, DL and ML classifiers are used for classification. Fan et al.^27^ employed a Dynamic Language Fusion (DLF) model by utilizing ResNet18 with Long Short-Term Memory (LSTM) for tumor evolution, BioGPT and Bidirectional Encoder Representations from Transformers (BERT) for semantic understanding, and cross-modal attention for interpretable feature fusion. Gomes and Barbosa^28^ developed DL models comprising CNN and ViT. Explainable AI (XAI) techniques such as Gradient-weighted Class Activation Mapping (Grad-CAM), Local Interpretable Model-agnostic Explanations (LIME), and Occlusion Sensitivity are also employed to ensure model interpretability and focus on tumor regions. Table 1 presents a comparative study of existing models based on their models, datasets, findings, and goals.

The limitations include difficulty with dataset discrepancy across MRI scanners and sequences. Additionally, massive labelled datasets are required for DL models, restricting applicability in resource-constrained settings. Furthermore, hybrid models integrating CNNs and ViT encounter high computational costs and feature redundancy, affecting efficiency. Moreover, XAI models are not effective with segmentation. Also, feature selection is improved by optimization techniques such as PSO. There also exists a research gap in presenting lightweight, interpretable models that maintain high accuracy across multimodal MRI without extensive pre-processing, while addressing it through adaptive attention mechanisms and hybrid learning strategies.

**Table 1:** Literature review of BT detection.

References	Models	Datasets	Findings	Goals
Tanone et al.^11^	Vision Transformer and CatBoost	Dual Open Dataset	Accuracy of 0.99316 and 0.90004.	The presented model is utilized to identify BT precisely and improve the classifier outcome.
Rajendirane et al.^12^	K-Means Clustering, MF	BRATS Dataset	Achieves a better outcome.	To develop a comprehensive method for improving BT identification by integrating advanced approaches.
Arshad Choudhry et al.^13^	AL-CAM, GCNN	Huge Dataset	Accomplishes an improved performance.	To project the explainability models in BT segmentation utilizing MRI and the utilization of conventional approaches offers interpretability tools.
Benedict et al.^14^	DWAE	BT Image Dataset	Achieves better accuracy.	To detect and localize tumor regions in the brain using sophisticated models and MRI.
Khan et al.^15^	CNN	MRI Dataset	Accuracy of 85%.	To tackle these concerns by utilizing data augmentation and a denoising model on clinical images, and concentrate on improving recognition efficacy.
Ali et al.^16^	GAN, TL, AE, DenseNet	BraTS 2021 Dataset	Dice score of 0.94.	To present a consistent and precise BT segmentation model for tackling challenges and offering effective tools for the treatment of brain diseases.
Allah et al.^17^	DCNN, Edge U-Net	BT Dataset	Dice score of 88.8%.	To present an approach for automated BT segmentation to resolve some of these concerns and manipulate to lessen background noise in the process of image.
Rahman and Islam^18^	PDCNN	Three Datasets	Accuracy of 97.33%, 97.60%, and 98.12%.	To develop an innovative model for eliminating both local and global attributes from dual parallel phases and managing overfitting.
Yadav et al.^19^	EfficientNetB7 Encoder, UNet + + Decoder, TL (AdvProp)	Kaggle LGG (110 Patients)	Dice of 0.9387, IoU of 0.9123	To precisely segment brain tumors for improved diagnosis and treatment planning.
Jenifer and Rajakumar^20^	HyperLink Bi-Attention TransUNet, Swin Transformer + CNN, Dual Bi-Attention (TRCA, CMRA), Disentanglement Learning Module, Retrieval-based Masked Autoencoder	BraTS 2023, REMBRANDT	Dice WT of 95.8%, TC of 95.6%, ET of 94.3%	To segment and classify multimodal MRI using a retrieval-based DL model.
Yadav, Kolekar, and Zope^21^	Mod-R2AU-Net, AG	MRI BT Images	Binary Accuracy: High, Dice: High, Iou: High	To segment BT from MRI scans using an attention-guided recurrent residual U-Net architecture.
Rastogi et al.^22^	CNN, MobileNet, NASNetMobile, ResNet101, RESUNET	TCGA-GBM (3,929 Images)	Accuracy of 0.950–0.970, Precision of 0.95–0.99, Recall of 0.92–0.99, F1-Score of 0.94–0.98	To automate the classification and localization of BT from MRI scans using a dual-stage DL.
Yadav et al.^23^	VGG-16	MRI BT Images	Accuracy of 98.5%, AUC of 99.0%, Recall of 98.4%, Precision of 98.3%, and F1-Score of 98.3%	To accurately classify BTs from MRI scans for improved diagnosis and treatment planning.
Yadav and Kolekar^24^	CNN, U-Net, AM	BraTS, FeTS, TCGA, Figshare	Accuracy: High, Dice: High, Iou: High, Hausdorff: Low	To review and analyze recent advancements in BT classification and segmentation.
Zhou et al.^25^	DL, RTDose, Transformer Models	MRI + RTDose, Brain Metastases	AUROC: 0.817, Subgroup AUROC: 0.723–0.843	To predict post-treatment BT recurrence using multimodal MRI and radiotherapy data.
Maheshwari, Kelkar, and Francis^26^	ResNet18 + EfficientNetB0, ViT, PSO, ACO, KNN, XGBoost	MRI BT Images	Accuracy of 99%, High Efficiency	To classify BTs from MRI images using a hybrid DL and optimization-based feature selection framework.
Fan et al.^27^	ResNet18 + LSTM, BioGPT, and BERT	10,287 MRI Images, 4 Public Datasets	Accuracy of 98.96%, Precision of 99.58%, AUC > 0.998	To classify BTs by integrating temporal MRI features and clinical text using a dynamic language fusion framework.
Gomes and Barbosa^28^	VGG-19, ResNet50, EfficientNetB3, Xception, MobileNetV2, DenseNet201, InceptionV3, ViT, Ensemble Modeling, Grad-CAM, LIME, Occlusion	Brain Tumor MRI (7,023 Images)	Accuracy of 89.47–98.17%, Best of 98.17%	To classify BTs from MRI scans using advanced DL and XAI techniques.

## Proposed methodology

**Fig. 1:**
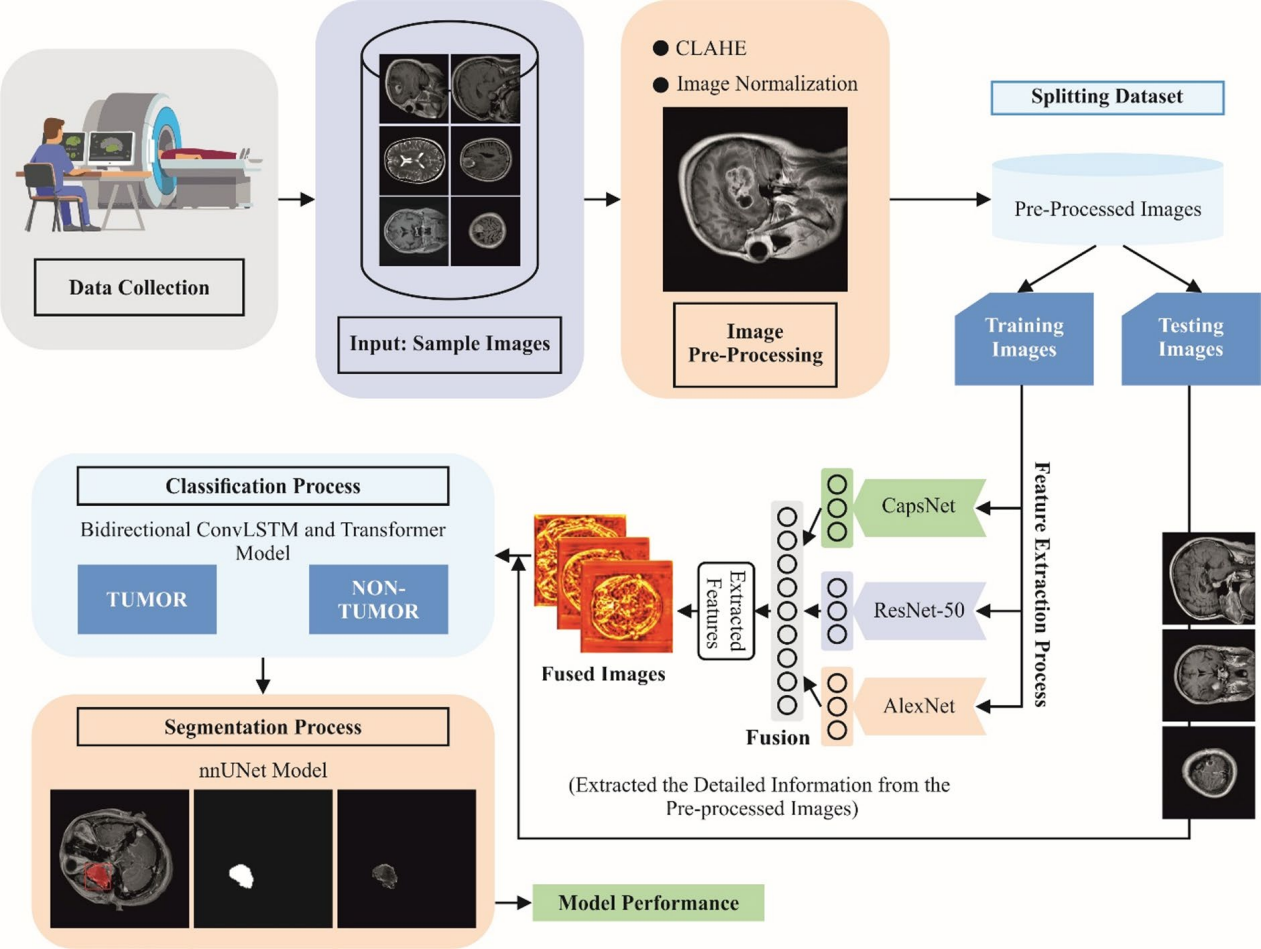
Overall block diagram of the MDFF-ABTDS approach.

In this study, the MDFF-ABTDS approach is proposed. This research aims to develop a multimodal DL model that incorporates feature fusion and a transformer for the accurate detection and segmentation of BTs in biomedical images. Figure 1 illustrates the complete workflow of the MDFF-ABTDS approach, which comprises several sequential stages essential for effective BT detection and segmentation. The detailed steps involved in this process are described below:

- Step 1: Input Image Pre-processing: Utilization of CLAHE and image normalization techniques to enhance contrast and standardize image intensity distributions, allowing effective feature extraction from heterogeneous MRI data.
- Step 2: Multimodal Feature Extraction: A multimodal CapsNet model, a ResNet-50 model, and an AlexNet method have been employed to capture local spatial details, hierarchical deep features, and capsule-based spatial relationships, improving the discriminative ability of extracted features.
- Step 3: Advanced Classification through Fusion Architecture: The integration of the (TBConvL-Net) model ensures both spatial and sequential feature understanding, thereby enhancing tumor classification accuracy.
- Step 4: Precise Tumor Segmentation: The nnUNet architecture is used for precise tumor localization and segmentation to ensure adaptability and high accuracy across diverse datasets.
- Step 5 Comprehensive Experimental Evaluation: Extensive experiments and comparative analyses demonstrate that the MDFF-ABTDS framework outperforms several existing models in terms of segmentation, dice coefficient, OSR, PPV, hit rate, f-statistics, and ROC-AUC.

### Image pre-processing methods

Initially, the image pre-processing is applied to CLAHE and image normalization for enhancing the contrast and quality of input images^29^. This is chosen for its efficiency in improving local contrast while also restricting noise amplification. CLAHE adapts contrast enhancement to small regions, unlike global histogram equalization, thus exhibiting robustness to illumination variations and preserving diagnostically crucial details. The pre-processing stage improves the input image for feature extraction. This stage certifies consistency and enhances image quality, tackling tasks related to fluctuating noise, imaging states, and restricted databases. The methods mentioned below are employed.

CLAHE: CLAHE was employed for enhancing the imaging quality, particularly in lower-contrast regions. The clip limit was computed below in the mathematical formulation.

1 $$\:{H}^{{\prime\:}}\left(i\right)=\mathrm{m}\mathrm{i}\mathrm{n}\left(H\left(i\right),\:clipLimit\right)$$

Once computing the Clip limit through Eq. (1), the function mapping computes the intensity for every pixel, improving local contrast as:

2 $$\:Output\left(i\right)=Max\:intensity\times\:\frac{Cumulative\:sum\:of\:{H}^{{\prime\:}}\left(i\right)}{Total\:Pixels\:}$$

Every tile was handled by Eq. (2), and the tiles are mixed to make the last equalized image. The training images are randomly selected from every class across numerous clip limits and tile grid sizes to aid the selection of CLAHE parameters.

Image normalization: Afterward, the normalization alters the pixel values in images to the same range, naturally, 0 to 1. For an 8-bit image, the maximal pixel value is 255. The formulation for image normalization is executed below.

3 $$\:Normalized\:Pixel=\frac{Pixel\:value}{255.0}$$

### Multimodal feature extraction

The multimodal techniques like CapsNet, ResNet-50, and AlexNet are used to extract the detailed information from the pre-processed imaging. Among the hybrid models, AlexNet shows excellence in capturing low-level texture and intensity discrepancies. Also, the deeper, high-level semantic features are effectively extracted by ResNet-50 using residual connections. Furthermore, multi-scale feature representation is ensured by the AlexNet and ResNet-50 models, and CapsNet additionally adds value by conserving spatial relationships between features through capsule vectors. The integration also enables better modelling of tumor shape, orientation, and part–whole relationships. Moreover, robustness is enhanced by CapsNet, unlike conventional CNNs that depend on pooling.

#### CapsNet model

The CapsNet is a variation of DL models, which is mainly presented for enhancing the efficiency of CNNs^30^. The CapsNet is appropriate for handling larger datasets. Moreover, the idea of the dynamic routing model and capsules was advanced for effective handling. CapsNet is nothing but a neural network that contains numerous capsules, where its input as well as output are signified as vectors. The dynamic routing model upgrades CapsNet among dual vector layers. The main notion of CapNet depended upon the standard of routing among capsules to permit the subsequent study of data. CapNet includes three unique layers: primary, upper, and the decoding network layers. The layer uses ReLu as an activation function. Then, it holds a stride of one, a kernel dimension of nine, and valid padding.

**Fig. 2:**
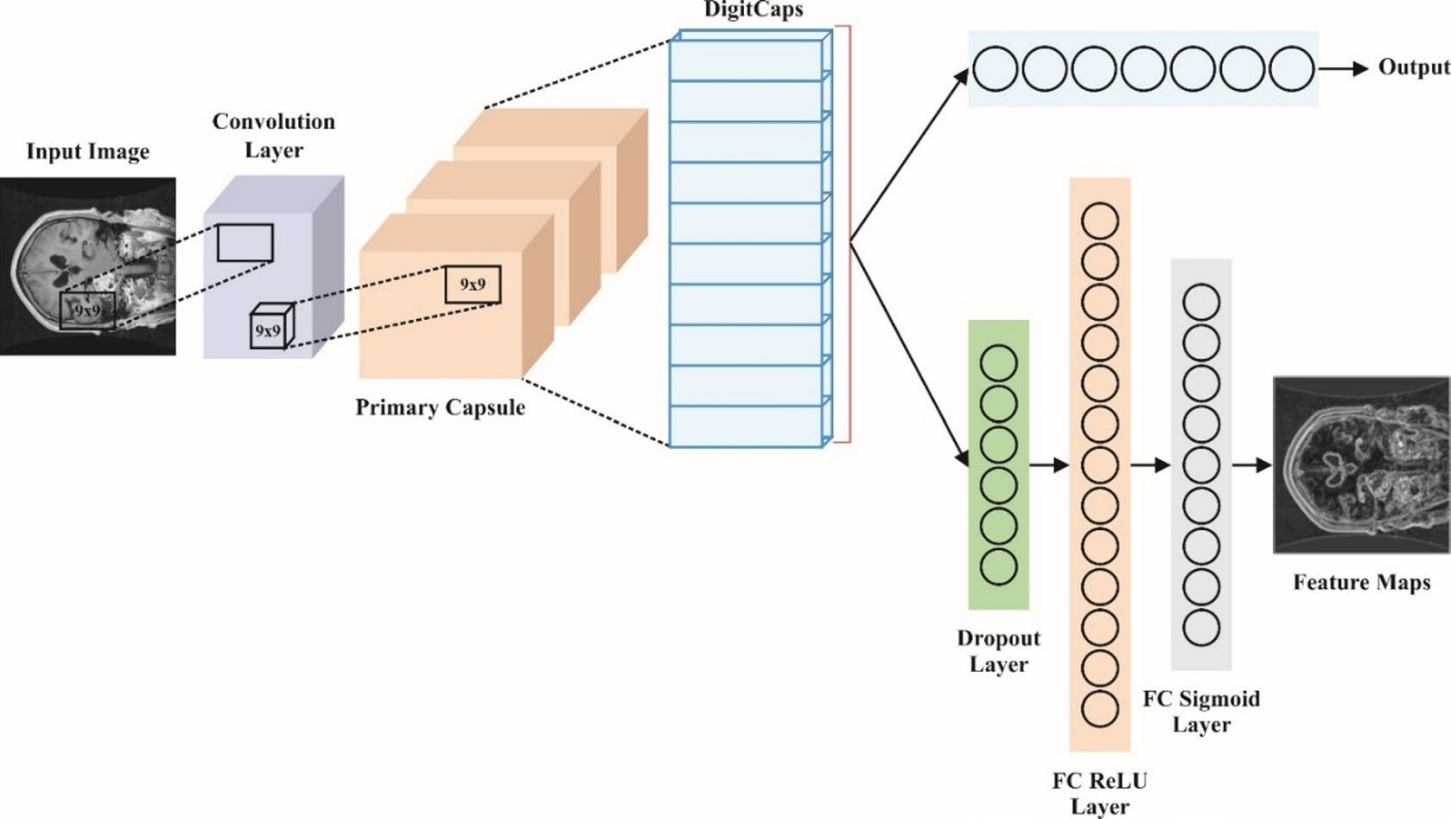
Structure of CapsNet.

The input database was initially transmitted to the convolutional layer, whereas the convolution procedure was performed on the data models. The following layer conveys an input to reform layers in the network that might be reflected and equivalent to 2-D convolutional layers. The main layer size is $\:32$x$\:8$ and holds a kernel dimension of nine, dual strides, and padding. The main layer mainly works for converting an input through a procedure called squash activation. A higher level of capsules was achieved in the last phase, which is also denoted as the secondary layer. In capsules, this layer mainly achieves active routing to get features and openly performs the routing function. The input was constantly focused and next handled over three completely interconnected decoding network levels. This system decodes the recognized features of every input, which leads to an effective identification. The backpropagation (BP) method was employed for enhancing the efficiency of the neural network throughout the training stage. Figure 2 portrays the infrastructure of CapsNet.

Capsules are groups of neurons that indicate dissimilar parameters for neurons. The drawback of CNNs is mainly related to the pooling layer. Thus, the layers are substituted in CapsNet by a suitable condition named ‘routing by agreement’. The output of the parent capsule was predicted, and the coefficients amongst these dual capsules are enlarged if this forecast aligns with the actual outcome of the parent capsules. Let $\:{u}_{j}$ be the capsule outcome $\:i$ and its prediction for parent $\:j$ capsule is calculated as

4 $$\:{u}_{j|i}={W}_{ij}{u}_{i}$$

whereas, $\:{u}_{j|i}$ signifies the vector prediction of the $\:jth$ outcome, $\:{W}_{ij}$ means the weight matrix. The coupling coefficients $\:{c}_{ij}$ are calculated utilizing the softmax function that is built upon the conformational level among the parent capsules and capsules beneath, depending upon the following formulation:

5 $$\:{c}_{ij}=\frac{\:\mathrm{e}\mathrm{x}\mathrm{p}\:\left({b}_{ij}\right)}{{\sum\:}_{k}\:\mathrm{e}\mathrm{x}\mathrm{p}\:\left({b}_{ij}\right)}$$

The log probability $\:{b}_{ij}$ specifies if the $\:ith$ capsule is linked to the$\:\:jth$ capsule, and is set to $\:0$ at the start of routing. Therefore, an input vector for parent $\:jth$ capsule was computed utilizing

6 $$\:{s}_{j}={\sum\:}_{i}{c}_{i{j}^{\mathcal{U}}j|i}$$

The following non-linear squash function averts any Capsules output vector and defines the last output according to its primary vector in Eq. (7).

7 $$\:{v}_{j}=\frac{\left|\right|{s}_{j}|{|}^{2}}{1+\left|\right|{s}_{j}|{|}^{2}\Vert\:{s}_{j}\Vert\:}$$

Here, $\:{s}_{j}$ is an input vector of $\:j$, and $\:{v}_{j}$ denotes the resultant vector of $\:j$. The log likelihoods should be studied according to concordance among $\:{v}_{j}$ and $\:{u}_{j}$, using the standard that a considerable inner product will result if the dual vectors agree.

8 $$\:{a}_{ij}={v}_{j}\bullet\:{u}_{j|i}$$

In the final layer, each capsule $\:k$ owns a loss function $\:{l}_{k}$ that allocates a major weight to capsules with protracted output. The loss function $\:{l}_{k}$ is calculated below using

9 $$\:{l}_{k}={T}_{k}\:\mathrm{m}\mathrm{a}\mathrm{x}\:(0,\:{m}^{+}-\Vert\:{v}_{k}\Vert\:)2+\lambda\:(1-{T}_{k})\:\mathrm{m}\mathrm{a}\mathrm{x}\:(0,\:\Vert\:{v}_{k}\Vert\:-{m}^{-}{)}^{2}$$

Here, $\:{T}_{k}$ equals 1 if $\:k$ exists; otherwise, $\:{T}_{k}$ equals $\:0.$ Prior to the learning procedure, the hyperparameters $\:{m}^{+},$ $\:{m}^{-}$, and $\:\lambda\:$ are stated.

#### ResNet-50 method

ResNet-50 is a 50‐layer CNN structure, which is commonly employed in numerous computer vision tasks^31^. The structure depends upon residual learning, which permits the system to study learning an unreferenced function. ResNet‐50 is a variation of the ResNet CNN with 50 layers. ResNet50 was formerly trained on ImageNet, which consists of a huge number of images. The significant layers of ResNet‐50, i.e., $\:3\mathrm{x}3$ conv 64, 128, 256, and 512, feature vector mapping, etc. The formulations for the ResNet‐50 method are defined utilising the below-mentioned Eq. (10).

10 $$\:f\left(X\right)=\sigma\:\left(W1\sigma\:\left(W1X+B1\right)+B2\right)$$

Here, $\:X$ denotes an input, $\:W1$ and $\:W2$ depict weight matrices, $\:B1$ and $\:B2$ illustrate bias terms, and $\:\sigma\:$ indicates a ReLU activation function. The residual connection is defined by Eq. (11).

11 $$\:f\left(X\right)+X$$

whereas $\:f\left(X\right)$ denotes an output of convolutional layers, and $\:X$ means an input. The following formulation defines the output value,

12 $$\:Output=Average\:Pooling\times\:Fully\:Connected\:Layer$$

where $\:Average\:Pooling$ denotes the average pooling layer, and $\:Fully\:Connected\:Layer$ means an FC layer. These calculations define the basic modules and architecture of the ResNet-50 method. The specific execution details might differ depending on the programming language and structure employed.

#### AlexNet model

For extracting significant features from data for brain tumour recognition, an architecture of the AlexNet CNN model has been proposed^32^. The foremost motive behind selecting the AlexNet-based CNN method for brain tumour recognition is its ease and ability to seize intricate spatial patterns in the dataset. Exact features of AlexNet, like its deep structure and convolutional layer, make it suitable for removing hierarchical attributes. The following are the reasons to choose the AlexNet-based CNN method: (1) When compared to other CNNs, the AlexNet offers a deep structure, enabling the seizure of more complex attributes. This enlarged depth authorizes the method for obtaining a hierarchical representation, which is vital for understanding the intricate patterns. (2) AlexNet integrates local response standardization by regularizing responses across numerous networks. This standardization device improves the model’s sturdiness to variants and helps in seizing relevant patterns. (3) In AlexNet, the convolutional filters used are mainly built to recognize spatial patterns at different scales. It is highly beneficial to take both global and local patterns, which are vital features for the precise classification of brain tumours.

AlexNet is nothing but a new CNN structure. It consists of eight layers, with three fully connected (FC) and five Conv. layers. The five convolution layers of the AlexNet CNN model are applied for extracting input image data. During the convolution layer, the size and number of filters differ. For deep feature study, the filter was relocated as an image. These three completely FC layers mitigate the dual-dimension feature matrix to 1D for forming the last layer of CNN models. Softmax layer assigns the class based on a probabilistic model, which is employed to complete the method.

### BT classification using fusion framework

In this step, these extracted features are then passed to a TBConvL-Net to classify tumours and non-tumours^33^. The spatial patterns are effectively captured by this model and compared to conventional models; TBConvL-Net gives robust feature fusion and better class separation while also mitigating data loss. The robustness and classification accuracy are also improved by the end-to-end learning capability, making it appropriate for reliable BT discrimination. Bi-directional LSTM acquires contextual data from past and upcoming stages in order. This can improve the capability of the network to learn intricate patterns and relations in data. Conversely, Swin Transformers (ST) employ a hierarchical model to process the non-overlapping local image patches, enabling them to learn attributes at numerous scales.

The output of batch normalisation $\:{\beta\:}_{N}^{out}$ is passed to the ConvLSTM layer. This layer contains an input, output gates, a memory cell $\:\left({M}_{{c}_{t}}\right)$, and a forgetting gate $\:\left({f}_{t}\right)$. These gates regulate modules for the layer of ConvLSTM, with the forget, input, and outcome gates particularly regulating the access, upgrade, and clear memory cells, correspondingly.

13 $$\:{M}_{{c}_{t}}={f}_{t}\otimes\:{M}_{{c}_{\left(t-1\right)}}+{i}_{t}\:\mathrm{t}\mathrm{a}\mathrm{n}\mathrm{h}\left({W}_{I,\:{M}_{c}}{I}_{t}+{W}_{h,\:{M}_{c}}{P}_{t-1}+{\beta\:}_{{M}_{c}}\right)$$

14 $$\:{\mathrm{o}}_{t}=\sigma\:\left({W}_{I,\:o}{I}_{t}+{W}_{h,o}{P}_{t-1}+{W}_{\:{M}_{c},o}\otimes\:{M}_{{c}_{t}}+{\beta\:}_{\mathrm{o}}\right)$$

15 $$\:{\mathrm{i}}_{t}=\sigma\:\left({W}_{I,\:i}{I}_{t}+{W}_{h,i}{P}_{t-1}+{W}_{\:{M}_{c},i}\otimes\:{M}_{{c}_{t}}+{\beta\:}_{\mathrm{i}}\right)$$

16 $$\:{\mathrm{f}}_{t}=\sigma\:\left({W}_{I,\:f}{I}_{t}+{W}_{h,f}{P}_{t-1}+{W}_{\:{M}_{c},f}\otimes\:{M}_{{c}_{t-1}}+{\beta\:}_{\mathrm{f}}\right)$$

17 $$\:{\mathrm{P}}_{t}={\mathrm{o}}_{t}\otimes\:\mathrm{t}\mathrm{a}\mathrm{n}\mathrm{h}\left({M}_{{c}_{t}}\right)$$

Here, $\:{I}_{t}$ and $\:{P}_{t}$ depict input and hidden tensors, $\:\otimes\:$ referring to Hadamard operations. 2-D convolution masks of input and hidden states are depicting $\:{W}_{I,\:*}$ and $\:{W}_{h,\:*}$. The biased terms of the memory cell, outcome, input, and forgetting gates were specified by $\:{\beta\:}_{{M}_{C}},{\beta\:}_{o},{\beta\:}_{i}$, and $\:{\beta\:}_{f}$, respectively.

In TBConvL-Net, BConvLSTM is employed, which expands classical ConvLSTM to acquire forward and backward dependency. This is beneficial, as understanding the past and upcoming context is vital to interpreting existing attributes of input. In BConvLSTM, input data has been processed in dual, distinct paths: forward and reverse directions. This aids in acquiring data from both the previous and succeeding frames regarding the existing input. The outcome of BConvLSTM has been calculated:

18 $$\:{I}_{out}=\mathrm{t}\mathrm{a}\mathrm{n}\mathrm{h}\left({W}^{(\to\:)}{P}_{t}^{\to\:}+{W}^{(\leftarrow\:)}{P}_{t}^{\leftarrow\:}+\beta\:\right)$$

Now $\:\beta\:$ indicates the bias elements and $\:{P}_{t}^{\to\:}$ and $\:{P}_{t}^{\leftarrow\:}$ imply the hidden state for forward and reverse directions, $\:\mathrm{t}\mathrm{a}\mathrm{n}\mathrm{h}$ signifies the hyperbolic tangent function, and is employed to non-linearly integrate the outcome of forward and reverse directions in BConvLSTM. This assures the incorporation of data from both directions and aids in acquiring intricate relations among the forward and reverse dependencies in input data.

Every patch that encapsulates a $\:4\times\:4$ pixel is seen as a “token”, its connected attributes being an integration of RGB pixel values. These attributes of raw value are specified by $\:d$, utilising a LE. Afterwards, a series of blocks of transformer, furnished with altered self-attention calculations, are implemented on these patch tokens. This enables the method to learn more intricate relations among input attributes that lead to enhanced outcomes on numerous tasks. This process results in a quartering of the token count that corresponds to downsampling of resolution2, with the dimension of output set to $\:2\times\:d$. This beginning phase of feature conversion and patch merging is specified as Block2. To increase the efficacy of the model, self‐attention has been implemented in local windows.

19 $$\:{C}_{MSA}=4(h\times\:w){d}^{2}+2(h\times\:w{)}^{2}d$$

and

20 $$\:{C}_{SW-MSA}=4(h\times\:w){d}^{2}+2{N}^{2}(h\times\:w{)}^{2}d$$

Here, the former has a quadratic relation to the count of patches and a linear relation for fixed $\:N$. This lighter version maintains the vital attributes of ST while lessening computation complexity.

21 $$\:{z}_{1}={\tau\:}_{in}\mathrm{\copyright\:}{L}_{N}\left(MSA\left({\tau\:}_{in}\right)\right)$$

Subsequently, the input $\:{z}_{2}$, $\:{\tau\:}_{out}$ to secondary STB has been evaluated as the concatenation of $\:{z}_{1}$, $\:{z}_{3}$, and the outcome of processing $\:{z}_{1}$, $\:{z}_{3}$ by $\:{L}_{N}$ and MLP as shown in Eqs. (22, 24).

22 $$\:{z}_{2}={z}_{1}\mathrm{\copyright\:}MLP\left({L}_{N}\left({z}_{1}\right)\right)$$

In the secondary STB, $\:{z}_{3}$ has been evaluated to concatenate $\:{z}_{2}$ with the outcome of the SW-MSA module after employing $\:{L}_{N}$:

23 $$\:{z}_{3}={z}_{2}\mathrm{\copyright\:}{L}_{N}\left(SW-MSA\left({z}_{2}\right)\right)$$

24 $$\:{\tau\:}_{out}={z}_{3}\mathrm{\copyright\:}MLP\left({L}_{N}\left({z}_{3}\right)\right)$$

### Segmentation model using nnUNet

At last, the tumor is categorized to detect its location using nnUNet for a precise segmentation process^34^. It utilizes the nnUNet framework, an extremely adaptive DL model particularly intended for segmentation. The nnUNet framework has depended upon FCN with an encoder-decoder framework.

The framework of nnUNet succeeds a U-shaped framework comprising a bottleneck, an encoder, and a decoder. It is intended to acquire a local and a global input image.

Encoder: It comprises a sequence of convolution layers succeeded by the operation of max-pooling. The convolution layer was activated utilizing the function of ReLU:

25 $$\:{x}_{l+1}=MaxPool\left(ReLU\left(Conv\left({x}_{l},\:{W}_{l}\right)\right)\right)$$

Here, $\:{W}_{l}$ signifies the weights of the convolution layer, and $\:{x}_{l}$ depicts the input to ‐th layer.

Bottleneck: bottleneck layer sits between the encoding and decoding, acquiring the most abstract attributes. It comprises convolution layers without pooling, assuring that theentire spatial resolution is preserved, while aiming at deep attributes.

Decoder: To upsample the mapping features back to the original image resolution.

26 $$\:{x}_{l-1}=ReLU\left(ConvTranspose\left({x}_{l},\:{W}_{l}\right)\right)$$

Skip connections were leveraged among the encoder and decoder’s equivalent layers to maintain spatial data, assisting in more accurate segmentation.

Output layer: The last resultant layer leverages the softmax activation function by creating the segmentation mask for every class:

27 $$\:P\left(classc\right)=\frac{\mathrm{exp}\left({z}_{c}\right)}{{\sum\:}_{{c}^{{\prime\:}}}\mathrm{e}\mathrm{x}\mathrm{p}\left({z}_{{c}^{{\prime\:}}}\right)}$$

Now $\:{z}_{c}$ signifies the outcome of the last convolution layer for class $\:c.$.

## Experimental result and analysis

The experimental validation of the MDFF-ABTDS method is investigated using the BT MRI dataset^35^ to validate its accuracy and robustness. The oversampling is used for handling data imbalance, and also, overfitting is mitigated using augmentation and dropout. Furthermore, data leakage is prevented by splitting training, validation, and test sets. The evaluation considers multiple performance metrics to measure the model’s segmentation and detection capabilities. The model is simulated using Python 3.6.5 on a PC with an i5-8600k, 250GB SSD, GeForce 1050Ti 4GB, 16GB RAM, and 1 TB HDD. Parameters include a learning rate of 0.01, ReLU activation, 50 epochs, 0.5 dropout, and a batch size of 5.

### Dataset used

**Fig. 3:**
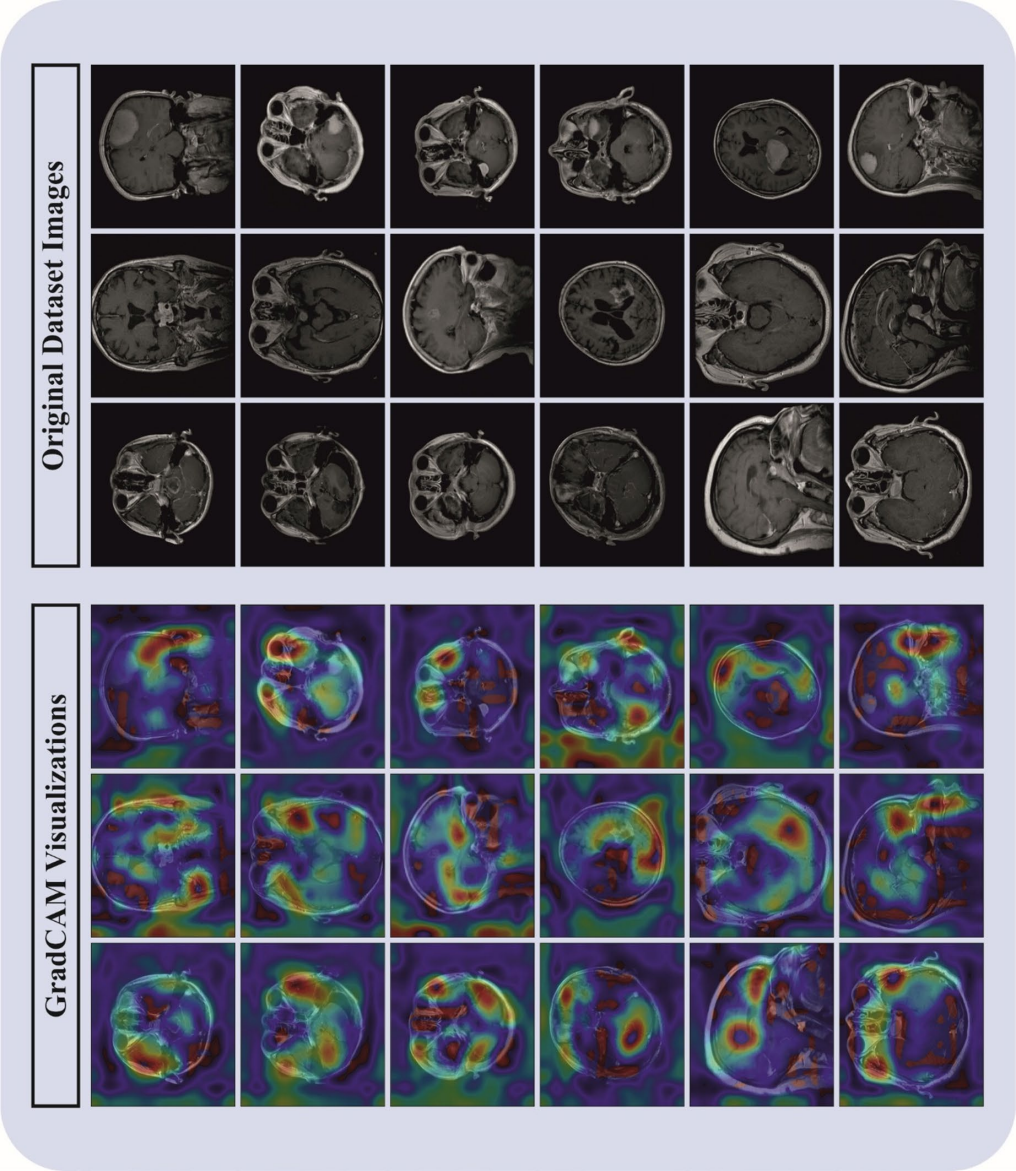
Sample images of original and GradCAM.

**Fig. 4:**
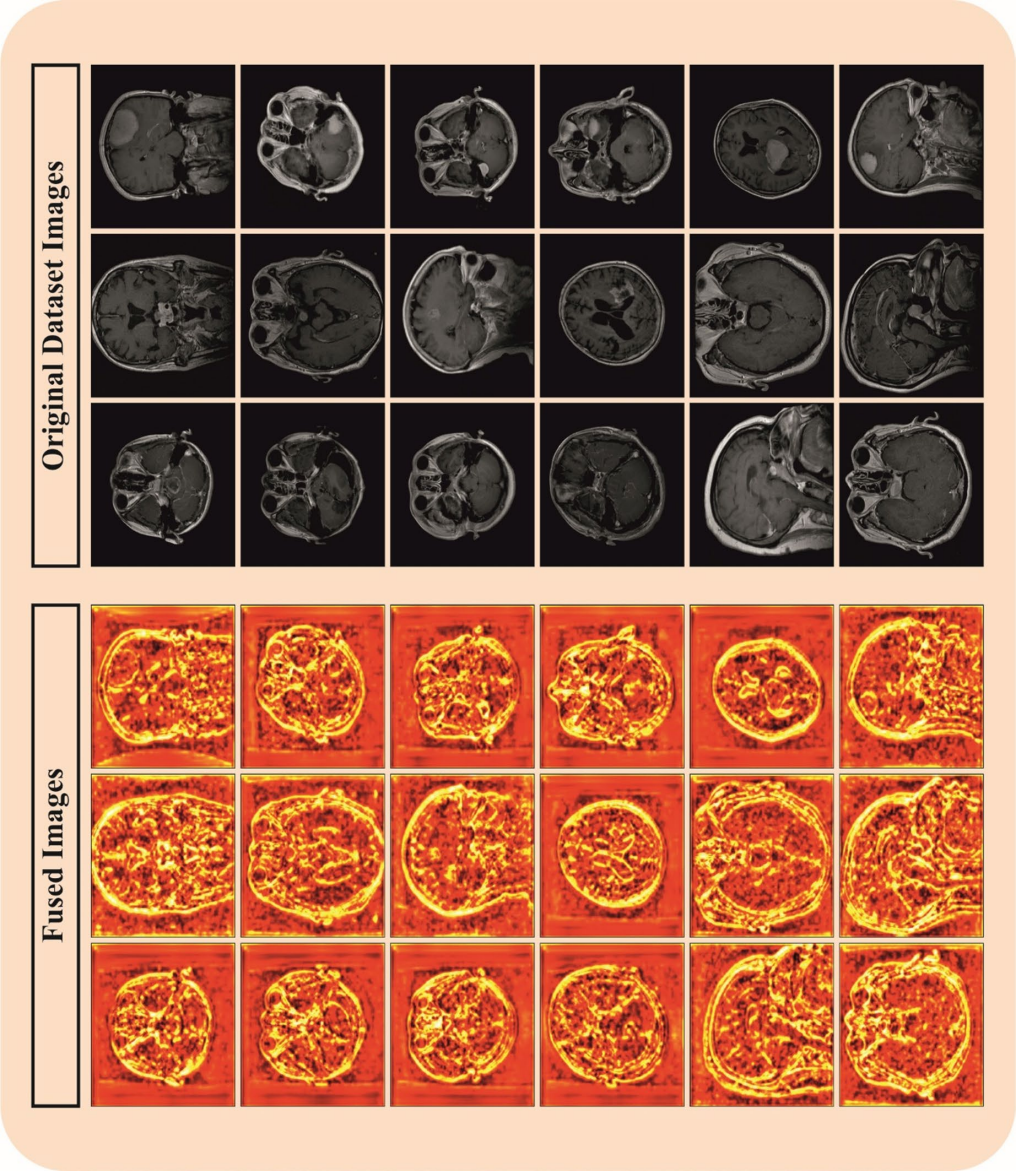
Sample images of the original and fused.

The MDFF-ABTDS method is examined under the BT MRI dataset. The dataset contains a total of 5,712 brain MRI images, classified into 4 various classes. The Glioma class involves 1,321 images, the Meningioma class consists of 1,339 images, while the Notumor class includes 1,595 images, making it the main category. In addition, the Pituitary class holds 1,457 images. This balanced distribution assures a different dataset appropriate for consistent BT analysis. Figure 3 defines the sample of original and gradCAM images. A few sample images of the original and fused images are demonstrated in Fig. 4.

### Performance measures

The efficacy of the model is calculated by employing conventional classification metrics attained from the confusion matrix. In the binary classifier problem, the confusion matrix creates 4 key outputs: True Positive (TP), False Positive (FP), True Negative (TN), False Negative (FN), Predicted Positives (PP), and Predicted Negatives (PN). These values are used from the matrix to calculate the execution of the model.

Accuracy (Overall Success Rate (OSR)) is an extensively utilized metric, and it is evaluated from the $\:TP,FP,FN$, and $\:TN.$

28 $$\:OSR\:=\frac{TP+TN}{TP+PP+PN+TN}$$

Precision (Positive predictive Value (PPV)): The percentage of properly recognized instances among all recognized instances.

29 $$\:PPV\:=\frac{TP}{TP+FP}$$

Recall (Hit Rate): The percentage of appropriately recognized instances among all actual instances.

30 $$\:\mathrm{H}\mathrm{i}\mathrm{t}\:\mathrm{R}\mathrm{a}\mathrm{t}\mathrm{e}\:=\frac{TP}{TP+FN}$$

The F-statistics is the harmonic mean of PR. The F-statistics range from a maximal value of one to a minimal value of zero.

31 $$\:\mathrm{F}-\mathrm{s}\mathrm{t}\mathrm{a}\mathrm{t}\mathrm{i}\mathrm{s}\mathrm{t}\mathrm{i}\mathrm{c}\mathrm{s}\:=2\cdot\:\frac{precision\cdot\:recall}{precision+recall}$$

**AUC (ROC-AUC)** is a widely utilized evaluation metric for ML problems that assesses the entire outcome of a classifier. In binary classification, the classifier predicts the probability of an instance being positive or negative, and the AUC measures the area under the ROC curve, plotting TPR against FPR.

32 $$\:TPR=\frac{TP}{TP+PN}$$

33 $$\:PPR=\frac{FP}{PP+TN}$$

34 $$\:AUC={\int\:}_{-{\infty\:}}^{{\infty\:}}[\frac{TP}{TP+FN}-\frac{PP}{FP+TN}]ds$$

### Confusion matrices

**Fig. 5:**
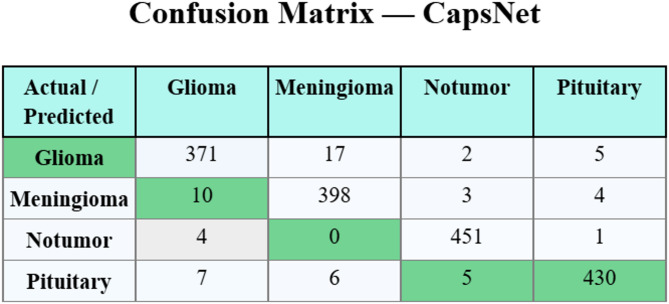
Confusion matrix of CapsNet.

**Fig. 6:**
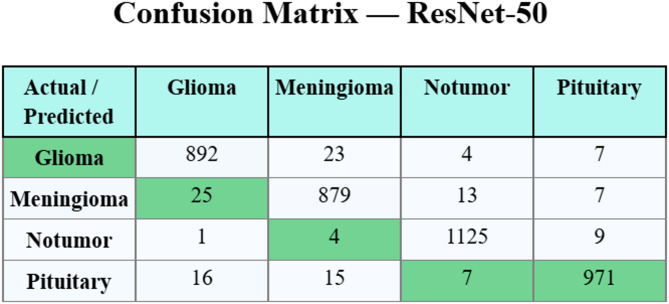
Confusion matrix of ResNet-50.

Figure 5 proves the confusion matrix of the CapsNet model. Next, Fig. 6 shows the confusion matrix of the ResNet-50 method. The result highlights effective classification and detection of each of the four classes.

**Fig. 7:**
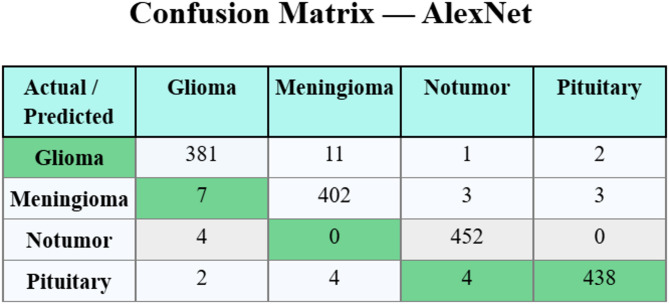
Confusion matrix of AlexNet.

**Fig. 8:**
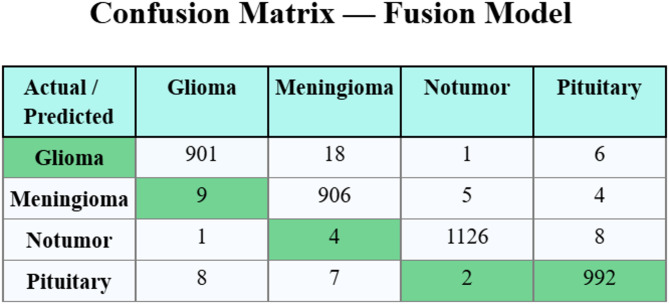
Confusion matrix of fusion model.

Figure 7 displays the confusion matrix of AlexNet. The outcome indicates that the approach has effective classification and detection of every four classes accurately. Also, Fig. 8 denotes the confusion matrix created by the fusion model. The results imply that the fusion technique has effective classification and detection of each 4 classes.

### Result analysis of training split

**Fig. 9:**
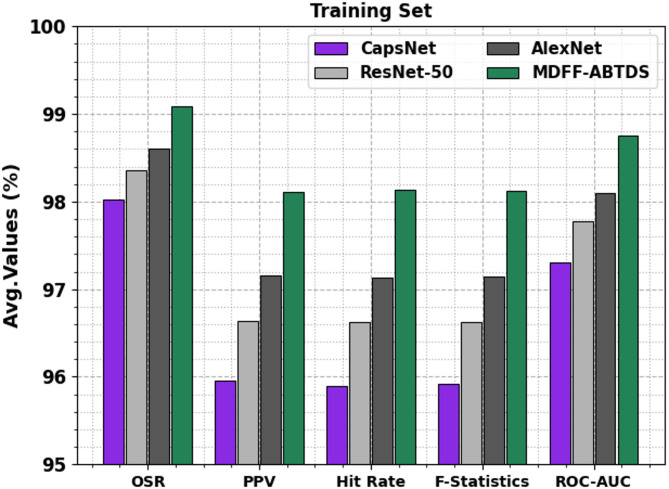
TRST outcome under various measures.

Table 2; Fig. 9 portray the training set (TRST) outcome under several measures. The CapsNet model obtains OSR, PPV, hit rate, F-statistics, and ROC-AUC of 98.02%, 95.96%, 95.90%, 95.92%, and 97.30%, respectively. Also, the ResNet-50 method attains OSR, PPV, hit rate, F-statistics, and ROC-AUC of 98.36%, 96.64%, 96.62%, 96.62%, and 97.77%, respectively. Besides, the AlexNet approach reaches OSR, PPV, hit rate, F-statistics, and ROC-AUC of 98.61%, 97.16%, 97.13%, 97.14%, and 98.10%, respectively. Likewise, the MDFF-ABTDS technique accomplishes OSR, PPV, hit rate, F-statistics, and ROC-AUC of 99.09%, 98.11%, 98.13%, 98.12%, and 98.76%, respectively.

**Table 2:** TRST outcome under various measures.

Training set					
Methods	OSR	PPV	Hit rate	F-statistics	ROC-AUC
CapsNet	98.02	95.96	95.90	95.92	97.30
ResNet-50	98.36	96.64	96.62	96.62	97.77
AlexNet	98.61	97.16	97.13	97.14	98.10
MDFF-ABTDS	99.09	98.11	98.13	98.12	98.76

### Result analysis of testing split

**Fig. 10:**
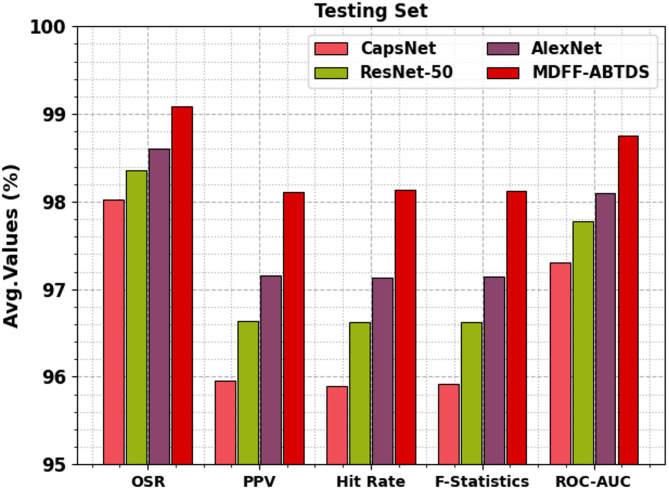
TRST outcome under various measures.

Table 3; Fig. 10 portray the training set (TRST) outcome under several measures. The CapsNet model obtains OSR, PPV, hit rate, F-statistics, and ROC-AUC of 98.02%, 95.96%, 95.90%, 95.92%, and 97.30%, respectively. Also, the ResNet-50 method attains OSR, PPV, hit rate, F-statistics, and ROC-AUC of 98.36%, 96.64%, 96.62%, 96.62%, and 97.77%, respectively. Besides, the AlexNet approach reaches OSR, PPV, hit rate, F-statistics, and ROC-AUC of 98.61%, 97.16%, 97.13%, 97.14%, and 98.10%, respectively. Likewise, the MDFF-ABTDS technique accomplishes OSR, PPV, hit rate, F-statistics, and ROC-AUC of 99.09%, 98.11%, 98.13%, 98.12%, and 98.76%, respectively.

**Table 3:** TRST outcome under various measures.

Testing set					
Methods	OSR	PPV	Hit rate	F-statistics	ROC-AUC
CapsNet	98.02	95.96	95.90	95.92	97.30
ResNet-50	98.36	96.64	96.62	96.62	97.77
AlexNet	98.61	97.16	97.13	97.14	98.10
MDFF-ABTDS	99.09	98.11	98.13	98.12	98.76

### Accuracy, loss, PR, and ROC curves

**Fig. 11:**
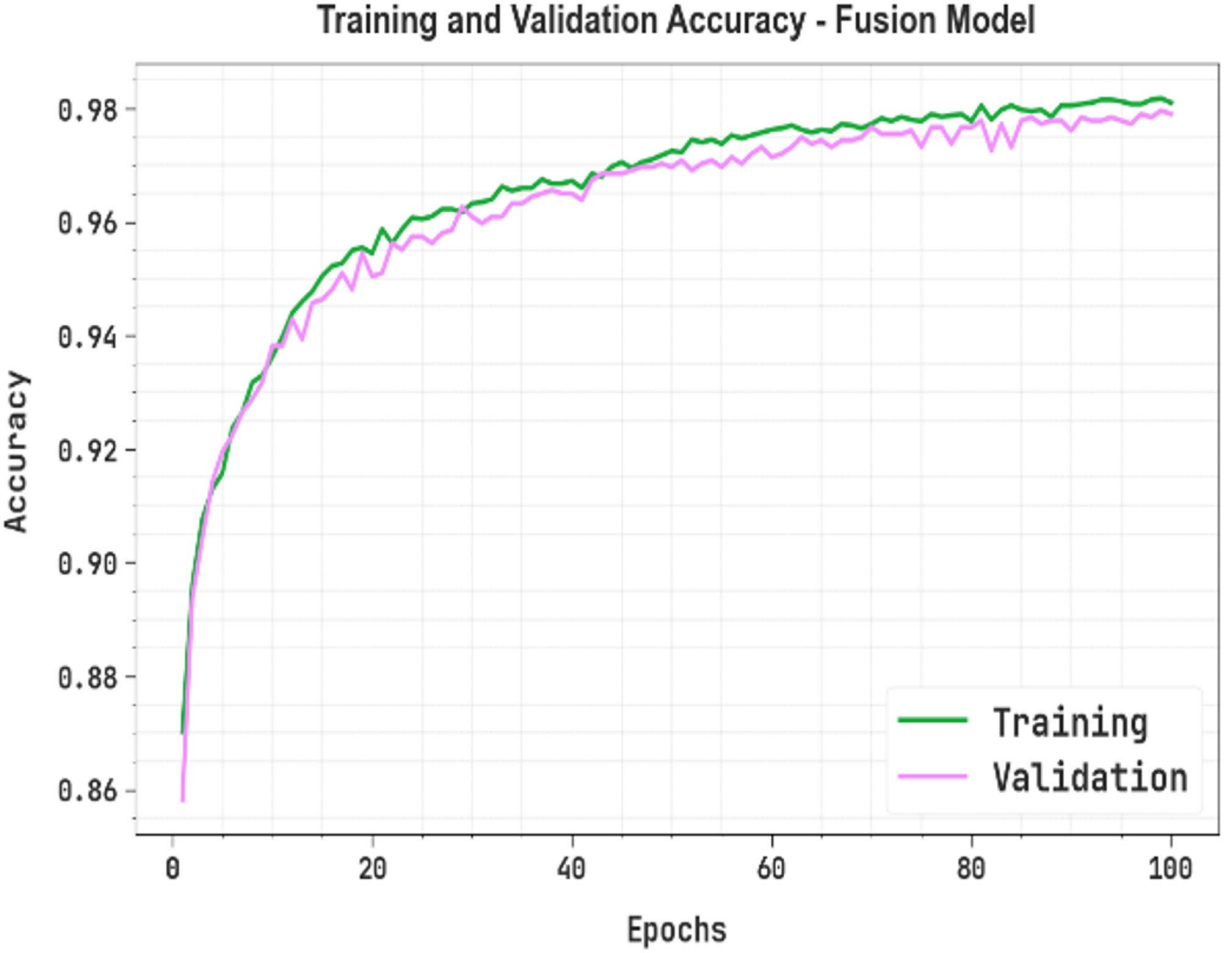
Accuracy curve of fusion model.

Figure 11 displays the training (TRAN) and validation (VALD) accuracy of the fusion model over 100 epochs. Both curves gradually rise and converge, showing effective learning. The VALD accuracy stays slightly above TRAN accuracy, indicating no over-fitting and good generalization.

**Fig. 12:**
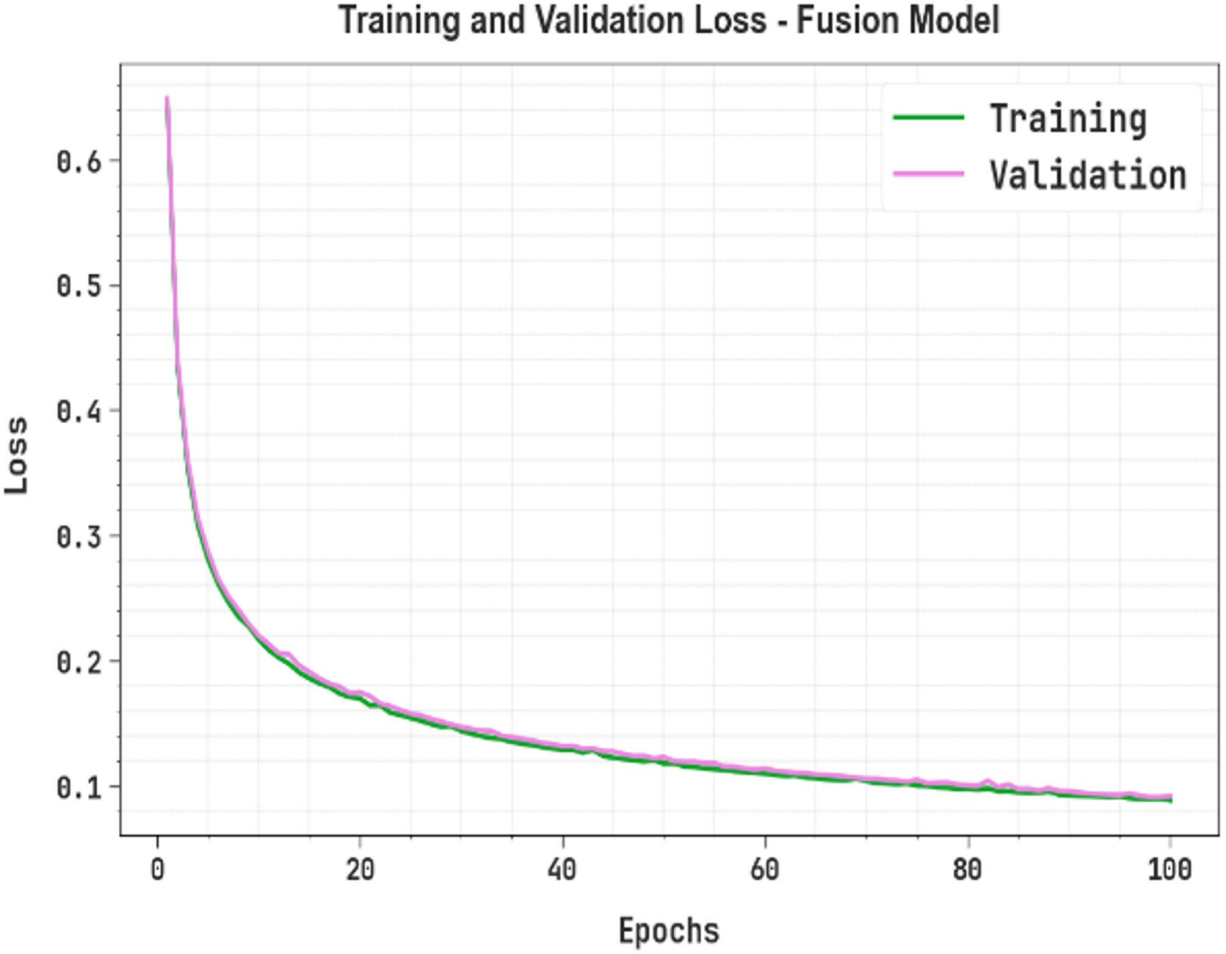
Loss curve of fusion model.

Next, Fig. 12 depicts the TRAN and VALD loss over 100 epochs. The VALD loss stays slightly inferior to the training loss through most epochs, hinting at good generalization and no signs of overfitting. Though fluctuations are seen, it is steady and more consistent.

**Fig. 13:**
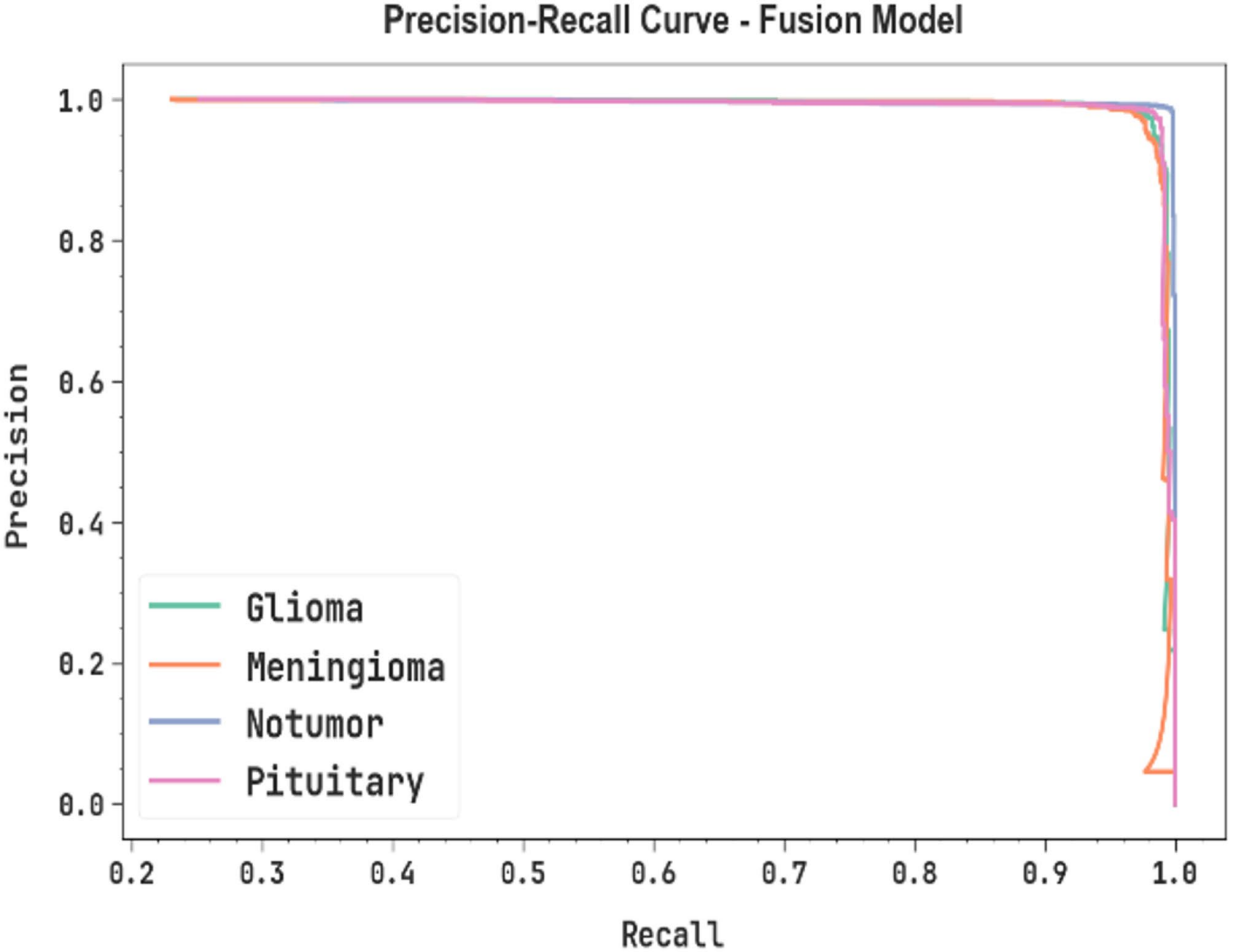
PR curve of fusion model.

In Fig. 13, the precision-recall (PR) curve investigation of the fusion technique is shown. The figure displays that the fusion model unceasingly achieves superior PR through numerous classes, demonstrating its capability of maintaining a considerable portion of true positive predictions among PR. The stable increase in PR results among each class describes the efficiency of the fusion model in the classification procedure.

**Fig. 14:**
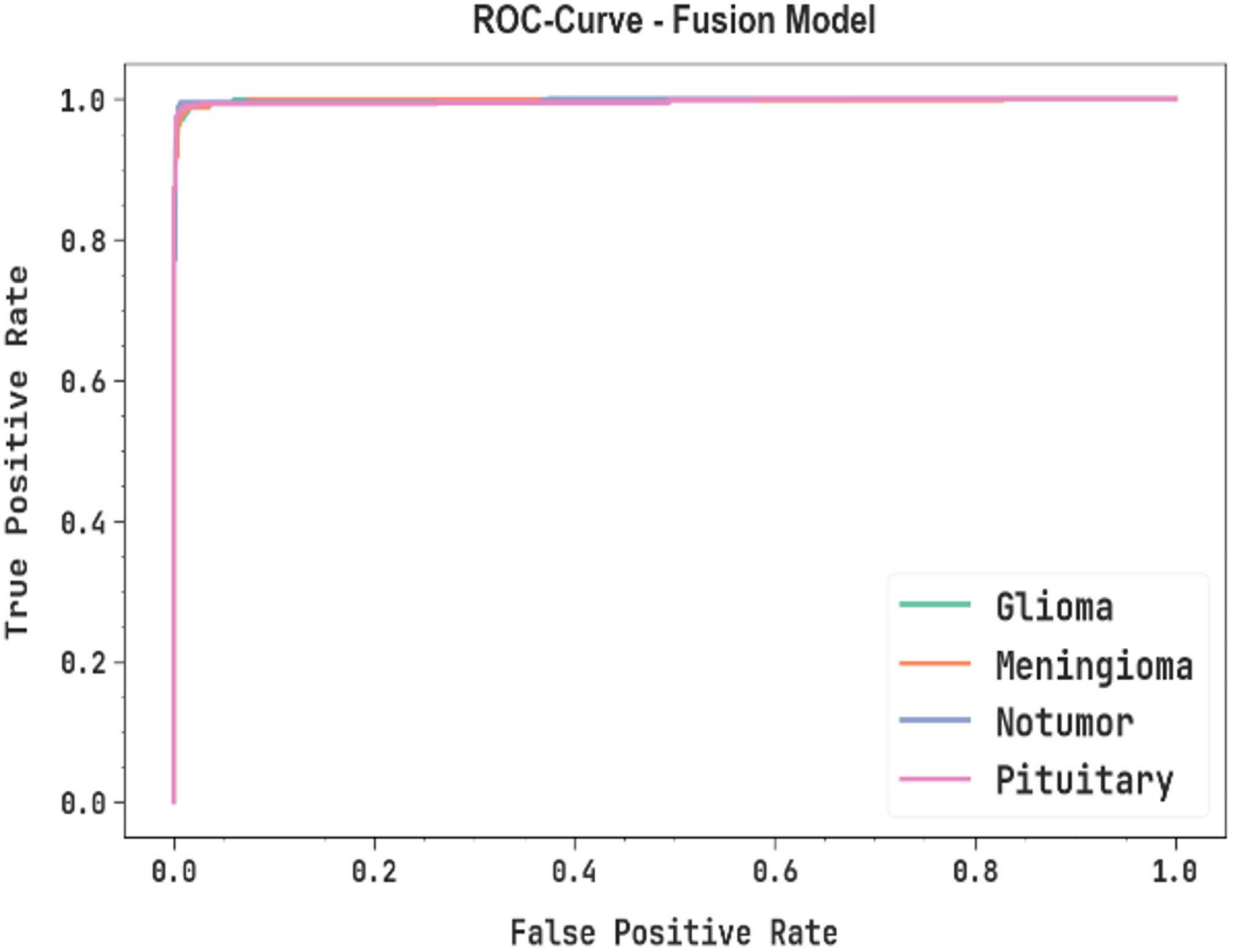
ROC curve of fusion model.

In Fig. 14, the ROC curve of the fusion model is shown. The performance suggests that the fusion model achieves improved ROC results across all classes, indicating an important ability to discriminate the classes.

### Comparative analysis with existing studies under diverse datasets

Table 4 portrays the comparative analysis of the MDFF-ABTDS methodology with different ML, DL, and fusion models under several measures like OSR, PPV, hit rate, F-statistics, and ROC-AUC. Also, this table offers an execution time outcome of the proposed and existing models^36–41^.

**Fig. 15:**
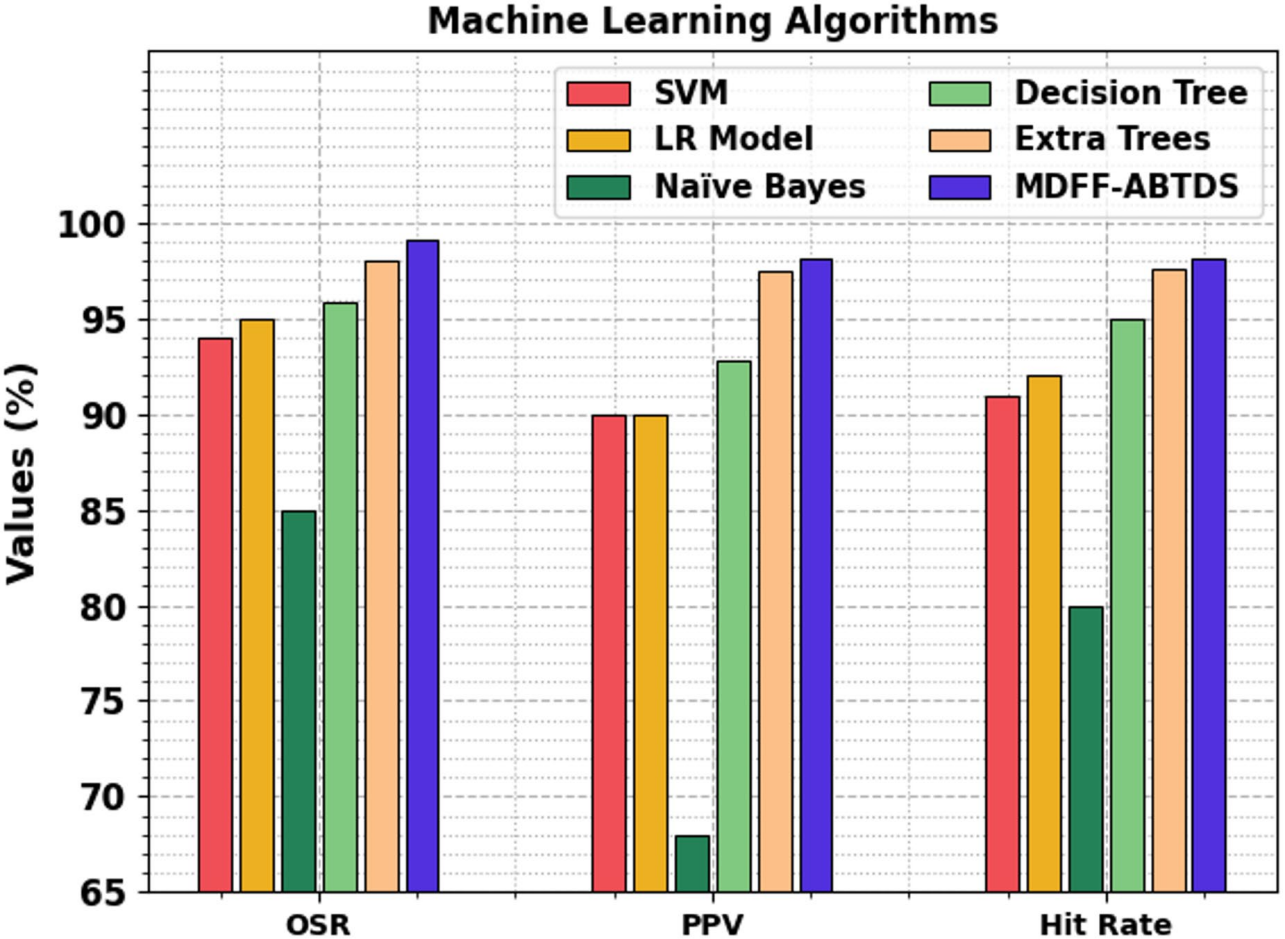
Comparative analysis of the MDFF-ABTDS model with DL approaches.

Figure 15 represents the performance analysis of the MDFF-ABTDS model with the ML approach. This analysis emphasizes that the MDFF-ABTDS method has achieved higher performance. Depending on $\:acc{ur}_{y}$, the MDFF-ABTDS method has attained a superior OSR of 99.09%. In contrast, the SVM, LR, Naïve Bayes (NB), Decision Tree (DT), and Extra Tree (ET) approaches have obtained lower OSR of 94.00%, 95.00%, 85.00%, 95.86%, and 98.00%, respectively. Likewise, based on hit rate, the MDFF-ABTDS method has attained a maximal hit rate of 98.13%, while the SVM, LR, NB, DT, and ET approaches have gained an inferior hit rate of 91.00%, 92.00%, 80.00%, 95.00%, and 97.59%, respectively.

**Fig. 16:**
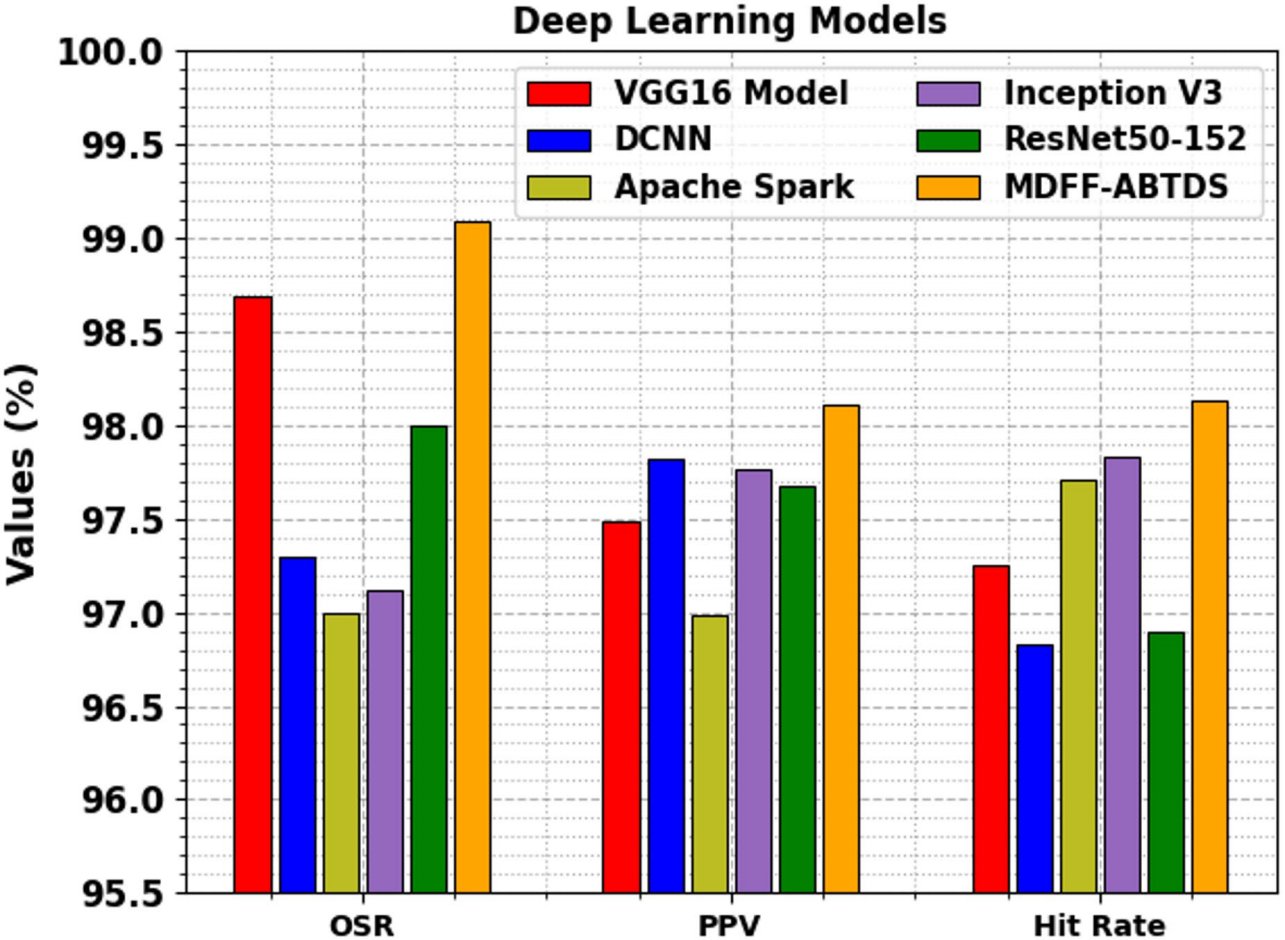
Comparative analysis of the MDFF-ABTDS model with DL approaches.

Figure 16 presents the comparative study of the MDFF-ABTDS method with DL methods. The results display that the MDFF-ABTDS model has reached higher performance with an OSR of 99.09%, a PPV of 98.11%, and a hit rate of 98.13%. Meanwhile, the DL techniques such as VGG16, DCNN, Apache Spark, Inception V3, and ResNet50-152 have obtained lower outcomes.

**Table 4:** Comparative outcome of MDFF-ABTDS approaches with existing models.

Architecture	OSR	PPV	Hit rate	Execution time (s)
With ML Models				
SVM	94.00	90.00	91.00	29.70
LR Model	95.00	90.00	92.00	19.16
NB	85.00	68.00	80.00	23.95
DT	95.86	92.78	95.00	9.86
ETs	98.00	97.44	97.59	16.11
With DL Models				
VGG16 Model	98.69	97.49	97.25	29.59
DCNN	97.30	97.82	96.83	24.39
Apache Spark	97.00	96.99	97.71	7.55
Inception V3	97.12	97.77	97.83	24.83
ResNet50-152	98.00	97.67	96.90	10.63
With Fusion Models				
Fusion-Brain-Net	93.32	93.76	91.37	7.55
Fusion-Brain-Net-fine-tuning	97.56	97.25	97.36	26.32
ResNet50 + CDBN	98.90	95.92	97.89	18.15
Fusion of CNNs	97.55	97.40	95.88	15.92
ResNet101 + CWAM	98.91	95.23	93.19	24.88
MDFF-ABTDS	99.09	98.11	98.13	4.07

**Fig. 17:**
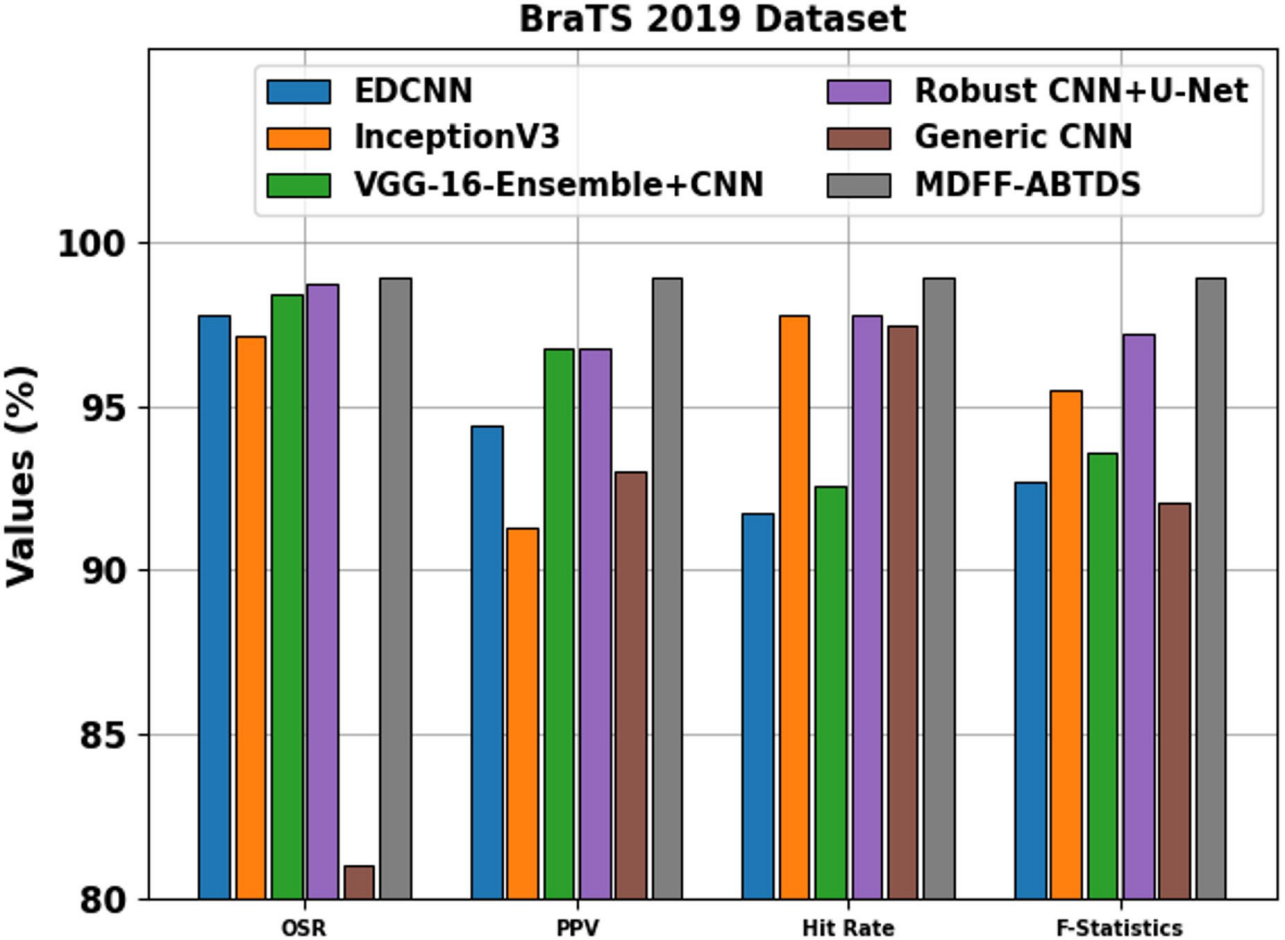
Comparison evaluation of the MDFF-ABTDS method with existing models.

Table 5; Fig. 17 specify the comparison evaluation of the MDFF-ABTDS method with existing models under the BraTS 2019 dataset^42,43^. The EDCNN model illustrates an OSR of 97.77%, PPV of 94.38%, Hit Rate of 91.72%, and F-Statistics of 92.69%. Furthermore, InceptionV3 attained an OSR of 97.12%, PPV of 91.31%, Hit Rate of 97.76%, and F-Statistics of 95.50%. Additionally, the VGG-16-Ensemble + CNN model depicted an OSR of 98.41%, PPV of 96.77%, Hit Rate of 92.56%, and F-Statistics of 93.57%, while the Robust CNN plus U-Net attained an OSR of 98.70%, PPV of 96.75%, Hit Rate of 97.79%, and F-Statistics of 97.18%. Furthermore, the Generic CNN method reached an OSR of 81.05%, PPV of 93.03%, Hit Rate of 97.42%, and F-Statistics of 92.09%. Finally, the MDFF-ABTDS model outperformed all existing methods with an OSR of 98.91%, PPV of 98.90%, Hit Rate of 98.90%, and F-Statistics of 98.93%, highlighting superior and well-balanced classification performance.

**Table 5:** Comparison evaluation of the MDFF-ABTDS method with existing models.

Models	OSR	PPV	Hit Rate	F-Statistics
EDCNN	97.77	94.38	91.72	92.69
InceptionV3	97.12	91.31	97.76	95.50
VGG-16-Ensemble + CNN	98.41	96.77	92.56	93.57
Robust CNN + U-Net	98.70	96.75	97.79	97.18
Generic CNN	81.05	93.03	97.42	92.09
MDFF-ABTDS	98.91	98.90	98.90	98.93

**Fig. 18:**
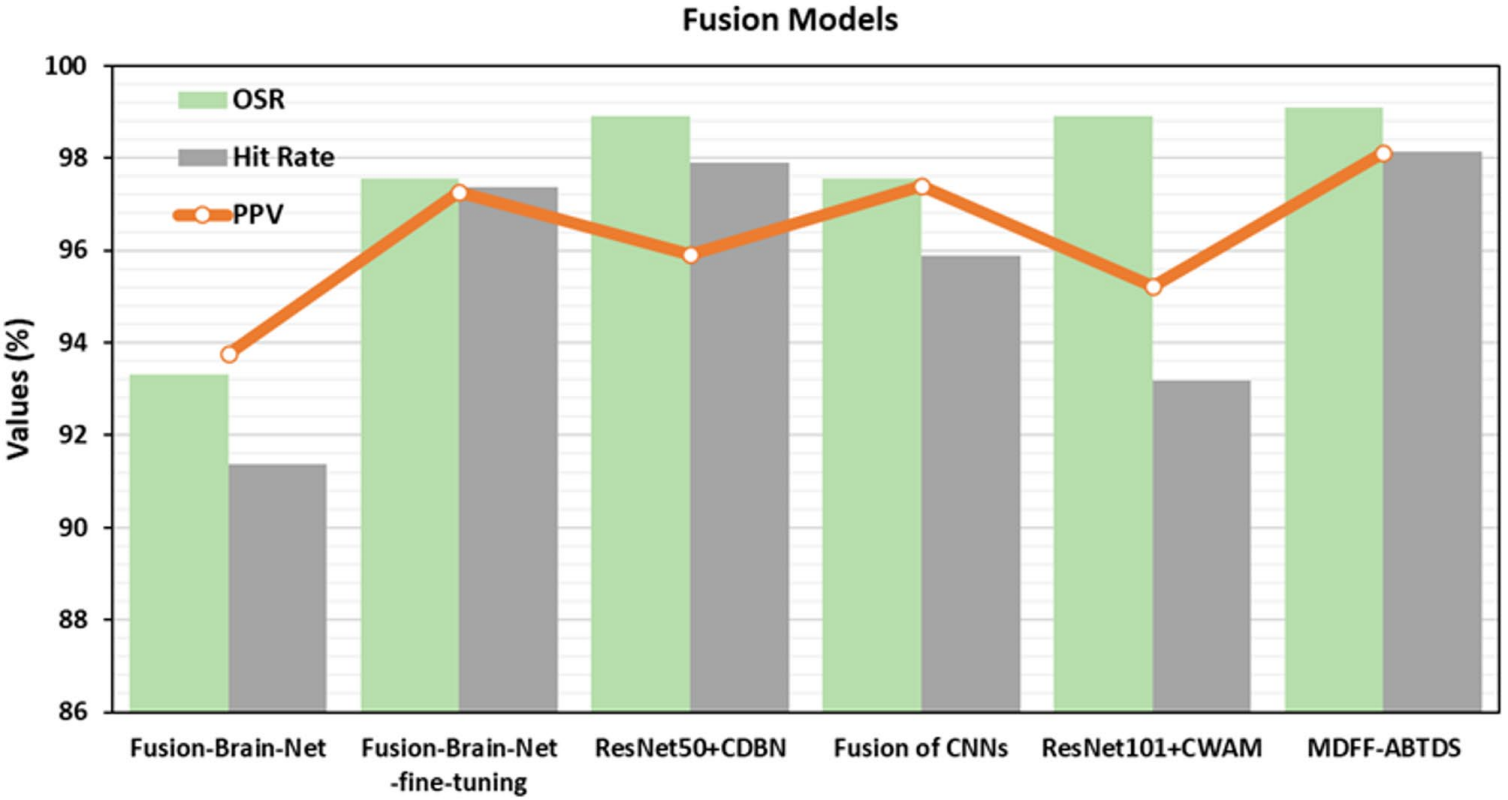
Comparative analysis of the MDFF-ABTDS model with fusion models.

The comparison analysis of the MDFF-ABTDS model with fusion models through three assessment metrics is shown below in Fig. 18. In contrast, the Fusion-Brain-Net method reveals relatively lesser OSR of 93.32%, PPV of 93.76%, and hit rate of 91.37%. Meanwhile, the Fusion-Brain-Net fine-tuning and Fusion of CNNs show slightly greater outcomes under three assessment metrics. The ResNet50 + CDBN approach has achieved reasonable performance with an OSR of 98.90%, a PPV of 95.92%, and a hit rate of 97.89%. Among the fusion models, the MDFF-ABTDS approach attains the greatest performance with an OSR of 99.09%, a PPV of 98.11%, and a hit rate of 98.13%.

**Fig. 19:**
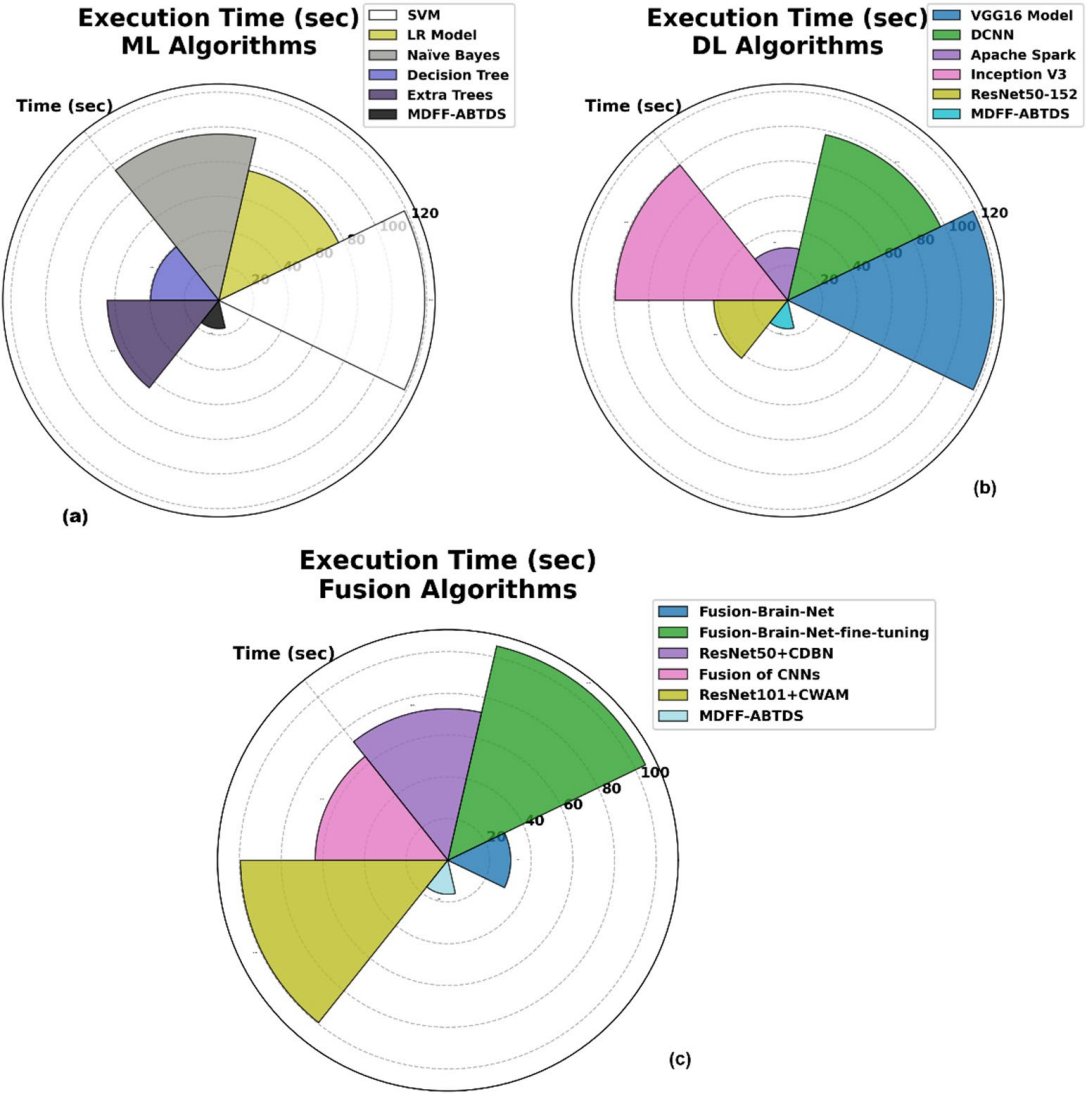
Execution time of MDFF-ABTDS model with ML, DL, and Fusion models.

Figure 19 exemplifies the execution time (ET) of the MDFF-ABTDS methodology with several ML, DL, and fusion approaches. Figure 18a displays the ET of the MDFF-ABTDS method with ML approach, conventional techniques like SVM, LR, NB, DT, and ETs reveal minimal execution times of 29.70 s, 19.16 s, 23.95 s, 9.86 s, and 16.11 s, while the MDFF-ABTDS method took a lower ET of 4.07 s. In Fig. 18b, the approaches such as VGG16, Inception V3, and ResNet50-152 display comparatively greater ET, whereas the MDFF-ABTDS method has an inferior ET of 4.07 s. Lastly, in the fusion models Fig. 18c, the MDFF-ABTDS method moderates ET relative to other fusion strategies, such as Fusion of CNNs and Fusion-Brain-Net fine-tuning, demonstrating its optimized structure for improved performance without extreme computational cost.

### Segmentation results

**Fig. 20:**
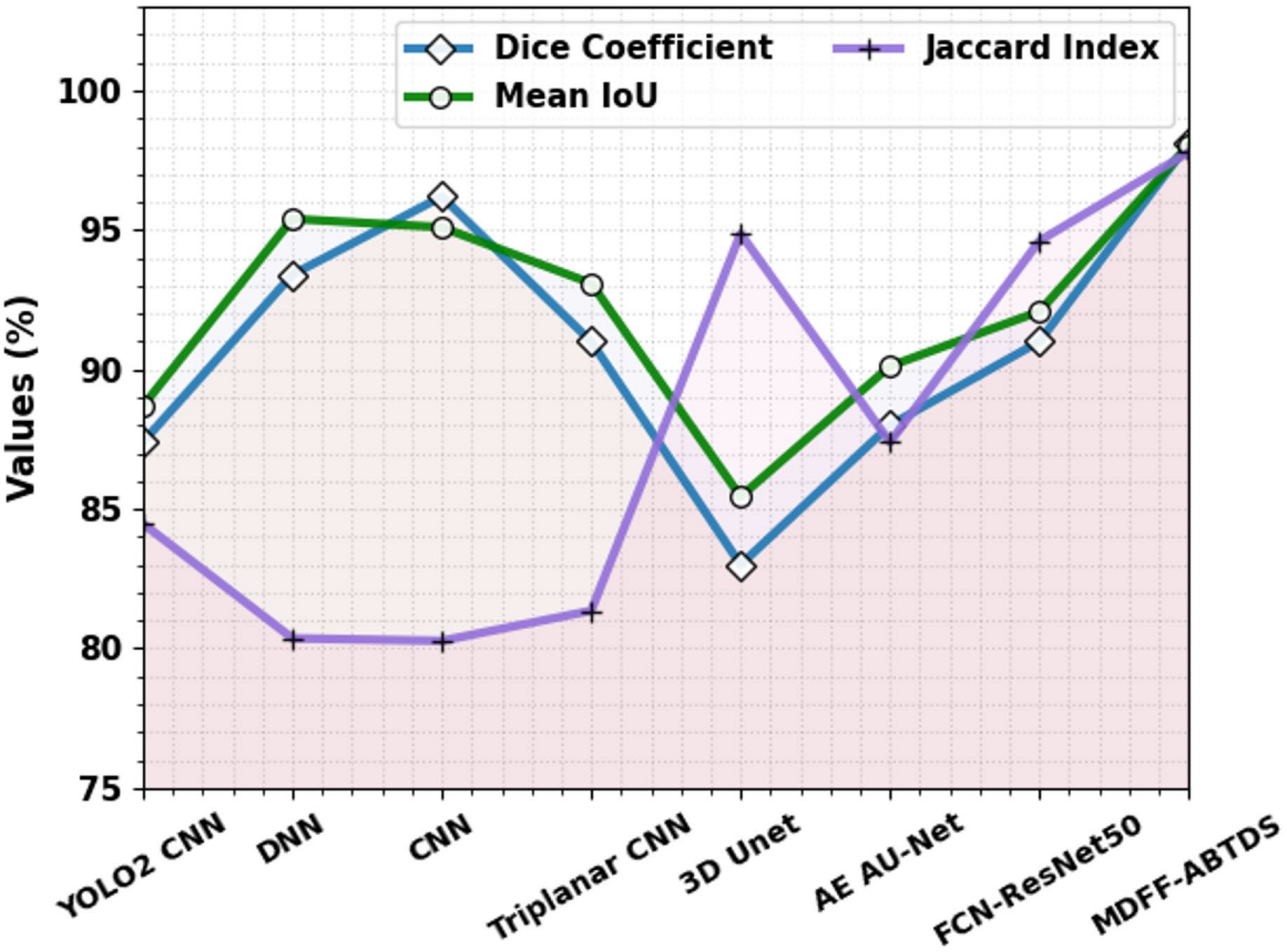
Segmentation outcome of the MDFF-ABTDS approach.

Table 6; Fig. 20 signify the segmentation result of the MDFF-ABTDS method with existing models^44,45^. The MDFF-ABTDS method has attained superior values with a dice coefficient of 98.11%, a mean IOU of 98.04%, and a Jaccard index of 97.79%. Meanwhile, the existing models, such as YOLO2 CNN, DNN, CNN, Triplanar CNN, 3D Unet, AE AU-Net, and FCN-ResNet50, have lower values. However, the MDFF-ABTDS technique is extremely effective for the BT classification and segmentation procedure.

**Table 6:** Segmentation outcome of the MDFF-ABTDS approach.

Approach	Dice coefficient	Mean IoU	Jaccard Index
YOLO2 CNN	87.40	88.70	84.47
DNN	93.40	95.40	80.36
CNN	96.20	95.10	80.27
Triplanar CNN	91.00	93.09	81.35
3D Unet	83.00	85.44	94.87
AE AU-Net	88.00	90.12	87.39
FCN-ResNet50	91.00	92.08	94.61
MDFF-ABTDS	98.11	98.04	97.79

### Ablation study analysis of the MDFF-ABTDS model

Table 7 represents the ablation study evaluation of the MDFF-ABTDS model. The ConvLSTM integrated with CapsNet, ResNet-50, and AlexNet attained the OSR values of 95.27%, 95.77%, and 96.52%. Also, Transformer-based models paired individually with CapsNet, ResNet-50, and AlexNet illustrate improved performance, achieving OSR values of 97.12%, 97.84%, and 98.59%, respectively. However, these configurations still lack comprehensive temporal modeling and feature fusion. The MDFF-ABTDS model, which integrates bidirectional ConvLSTM and Transformer with fused feature representations, achieves the highest OSR of 99.09%, PPV of 98.11%, and Hit Rate of 98.13%, clearly emphasizing superior classification performance.

**Table 7:** Ablation study evaluation result of the MDFF-ABTDS model.

Models	OSR	PPV	Hit Rate
ConvLSTM+CapsNet (Without Bidirectional, Transformer, ResNet-50, and AlexNet)	95.27	94.24	94.02
ConvLSTM+ResNet-50 (Without Bidirectional, Transformer, CapsNet, and AlexNet)	95.77	94.81	94.56
ConvLSTM+AlexNet (Without Bidirectional, Transformer, ResNet-50, and CapsNet)	96.52	95.47	95.09
Transformer+ CapsNet (Without Bidirectional and ConvLSTM and ResNet-50 and AlexNet)	97.12	96.27	95.77
Transformer+ResNet-50 (Without Bidirectional, Transformer, CapsNet, and AlexNet)	97.84	96.88	96.55
Transformer+AlexNet (Without Bidirectional, Transformer, ResNet-50, and CapsNet)	98.59	97.52	97.34
MDFF-ABTDS (Bidirectional ConvLSTM and Transformer with feature fusion models)	99.09	98.11	98.13

### Computational efficiency evaluation of the MDFF-ABTDS method

Table 8 depicts the evaluation of the MDFF-ABTDS method in terms of Floating-Point Operations (FLOPs), Graphics Processing Unit (GPU), and inference time^46^. The MDFF-ABTDS method achieved a FLOPs of 0.06 G, GPU usage of 1532 M, and inference time of 1.03 ms. Furthermore, the Input Cascaded CNN recorded a FLOPs of 20.04 G, GPU usage of 5176 M, and inference time of 4.05 ms. Additionally, Pretrained VGG-19 attained a FLOPs of 0.33 G, GPU usage of 5840 M, and inference time of 4.70 ms. Moreover, the ResNet101 model attained a FLOPs of 45.70 G, GPU usage of 3231 M, and inference time of 3.93 ms. Also, the VGG16 approach reached a FLOPs of 7.76 G, GPU usage of 3574 M, and inference time of 5.46 ms. Likewise, GoogleNet achieved a FLOPs of 41.20 G, GPU usage of 3116 M, and inference time of 2.78 ms. Finally, Fine-tuned EfficientNet-V2S recorded a FLOPs of 31.00 G, GPU usage of 3777 M, and inference time of 5.40 ms, thus highlighting the lightweight, fast, and superior performance of the MDFF-ABTDS model, requiring minimal GPU resources.

**Table 8:** Evaluation of the MDFF-ABTDS method in terms of FLOPs, GPU, and inference time.

Models	FLOPs (G)	GPU (M)	Inference Time (ms)
Input Cascaded CNN	20.04	5176	4.05
Pretrained VGG-19	0.33	5840	4.70
Resnet101	45.70	3231	3.93
VGG16	7.76	3574	5.46
GoogleNet	41.20	3116	2.78
Fine-tuned EfficientNet-V2S	31.00	3777	5.40
MDFF-ABTDS	0.06	1532	1.03

## Conclusion

In this manuscript, the MDFF-ABTDS approach is proposed for BT segmentation and classification. This objective aims to develop a multimodal DL technique that integrates feature fusion and transformer networks for the precise detection and segmentation of BTs in medical images. Initially, the MDFF-ABTDS approach executed image pre-processing using CLAHE and image normalization to increase the contrast and quality of input images for effective analysis. Feature extraction is carried out through fusion models, namely CapsNet, ResNet-50, and AlexNet, to extract the detailed information from the pre-processed images. These extracted features are then passed to a TBConvL-Net approach to classify tumors and non-tumors effectively. Finally, the tumor is classified to identify its location using the nnUNet method for a precise segmentation process. A series of experimental analyses of the MDFF-ABTDS method portrayed a superior accuracy value of 98.91% over existing models under the BT MRI dataset.

## Data Availability

The data that support the findings of this study are openly available in the Kaggle repository at https://www.kaggle.com/datasets/masoudnickparvar/brain-tumor-mri-dataset/data, https://www.kaggle.com/datasets/aryanfelix/brats-2019-traintestvalid/data, reference numbers^35,42^.
